# Safety and Immunogenicity of EBA-175 RII-NG Malaria Vaccine Administered Intramuscularly in Semi-Immune Adults: A Phase 1, Double-Blinded Placebo Controlled Dosage Escalation Study

**DOI:** 10.1371/journal.pone.0163066

**Published:** 2016-09-19

**Authors:** Kwadwo A. Koram, Bright Adu, Josephine Ocran, Yaa S. Karikari, Susan Adu-Amankwah, Michael Ntiri, Benjamin Abuaku, Daniel Dodoo, Ben Gyan, Karl C. Kronmann, Francis Nkrumah

**Affiliations:** 1 Department of Epidemiology, Noguchi Memorial Institute for Medical Research, College of Health Sciences, University of Ghana, Legon, Ghana; 2 Department of Immunology, Noguchi Memorial Institute for Medical Research, College of Health Sciences, University of Ghana, Legon, Ghana; 3 US Naval Medical Research Unit 3, Ghana Detachment, Cantonments, Accra, Ghana; Johns Hopkins Bloomberg School of Public Health, UNITED STATES

## Abstract

The erythrocyte binding antigen region II (EBA-175 RII) is a *Plasmodium falciparum* ligand that mediates erythrocyte invasion and is considered an important malaria vaccine candidate. A phase Ia trial in malaria naïve adults living in the United States found the recombinant non-glycosylated vaccine antigen, EBA-175 RII-NG adjuvanted with aluminium phosphate to be safe, immunogenic and capable of inducing biologically active antibodies that can inhibit parasite growth *in vitro*. The aim of the current study was to assess the safety and immunogenicity of this vaccine in malaria exposed semi-immune healthy adults living in a malaria endemic country, Ghana. In this double-blinded, placebo controlled, dose escalation phase I trial, eighteen subjects per group received ascending dose concentrations (5 μg, 20 μg or 80 μg) of the vaccine intramuscularly at 0, 1 and 6 months, while 6 subjects received placebo (normal saline). The primary end point was the number of subjects experiencing Grade 3 systemic or local adverse events within 14 days post-vaccination. Serious adverse events were assessed throughout the study period. Blood samples for immunological analyses were collected at days 0, 14, 28, 42, 180 and 194. A total of 52 subjects received three doses of the vaccine in the respective groups. No serious adverse events were reported. The majority of all adverse events reported were mild to moderate in severity, with local pain and tenderness being the most common. All adverse events, irrespective of severity, resolved without any sequelae. Subjects who received any of the EBA-175 RII-NG doses had high immunoglobulin G levels which moderately inhibited *P*. *falciparum* growth *in vitro*, compared to those in the placebo group. In conclusion, the EBA-175 RII-NG vaccine was safe, well tolerated and immunogenic in malaria semi-immune Ghanaian adults. Its further development is recommended.

**Trial registration:**

ClinicalTrials.gov. Identifier: NCT01026246

## Introduction

Malaria accounts for nearly half a million deaths annually, mostly in children under the age of 5 years and pregnant women living in economically developing countries where an estimated 5% of the gross domestic product is consumed by the direct and indirect health costs of malaria [1]. It is expected that a highly effective malaria vaccine in addition to the current malaria intervention measures would greatly reduce the global burden of malaria, however, such a vaccine has so far remained elusive. The recently licensed malaria vaccine, RTS,S/AS01, under the trade name Mosquirix, provides only about 20 to 50% protection against malaria [2], underscoring the need for intensified efforts towards obtaining a more efficacious vaccine.

Currently, two broad categories of vaccines against malaria are being pursued: disease-modifying and infection-preventing, although lately with the drive towards malaria elimination and eradication, attention is also being turned to vaccines that interrupt transmission [3, 4]. Disease-modifying vaccines are designed to reduce mortality and severe disease by targeting mainly the erythrocytic stages of the parasite [5–15]. Infection-preventing vaccines, however, are meant to prevent malaria infection and are usually targeted at the pre-erythrocytic stages of the parasite lifecycle [2, 16, 17]. Vaccines against the sexual stages of the parasite are intended to interrupt transmission at the population level and are being increasingly considered as an option for malaria elimination and eradication [18].

In endemic populations, naturally acquired partial immunity to malaria is common among adults but the immunological targets and mechanisms involved remain poorly understood, thus hampering malaria vaccine development. Ligands used by malaria parasites for erythrocyte invasion may be important targets of malaria protective antibodies, though *P*. *falciparum* is known to use multiple invasion pathways and vary its expression of erythrocyte-binding antigenic proteins and reticulocyte-binding protein homologues in order to evade host immune responses [19–21]. Notwithstanding, crucial invasion ligands may serve as composites of multi-epitope, ligand-blocking blood-stage vaccines against malaria [22].

The 175 KDa-erythrocyte binding antigen (EBA-175) is a parasite ligand that binds sialic acid residues of glycophorin A on the surface of the erythrocyte during the invasion process [19, 23], and is considered a leading malaria vaccine candidate. Sialic acid binding is largely limited to the highly conserved Region II (RII) of EBA175 [19, 24], but other regions have also been recently shown to interact with glycophorin A [25]. Antibodies against EBA-175 RII block erythrocyte invasion of both sialic acid-dependent and sialic acid-independent (alternative invasive pathway) parasite strains *in vitro* [22]. Aotus monkeys immunized with EBA-175 RII showed significantly decreased parasitaemia compared to the control group in challenge experiments [26].

The non-glycosylated vaccine antigen, EBA-175 RII-NG, expressed in the methylotrophic yeast, *Pichia pastoris* and adjuvanted with aluminium phosphate (Adju-Phos^®^) was safe and immunogenic in a phase I study among malaria-naïve adults in the United States. Significant *in-vitro* inhibitory activity on blood stage *P*. *falciparum* growth was observed with sera from study participants [27].

Here, we report the findings of a phase I trial of the EBA-175 RII-NG vaccine candidate in healthy, malaria exposed semi-immune adults living in malaria endemic region, Ghana, to assess safety and immunogenicity to ascending doses of the vaccine.

## Materials and Method

The study protocol and supporting CONSORT checklist are available as supporting information; see S1 Protocol and S1 CONSORT Checklist.

### Study design and ethics

The study was a phase I randomized double-blind dose escalation trial which was carried out from June 2010 to March 2012 to assess safety, reactogenicity and immunogenicity of EBA-175 RII-NG vaccine candidate. Subjects were randomized (9:1 ratio) to receive three doses of EBA-175 RII-NG or placebo (normal saline) via the intramuscular route. Eighteen (18) subjects per cohort received EBA-175 RII-NG at each of the following dosage levels: 5μg (Cohort A), 20μg (Cohort B), and 80 μg (Cohort C) with 500 μg of Adju-Phos^®^ adjuvant while 2 subjects in each cohort received placebo. These vaccine concentrations were chosen based on results from the phase I study conducted in the healthy malaria-naïve adult subjects in the US [27]. In the US study, the 160 μg vaccine dose had similar immunogenicity to the 80 μg dose and hence the current study did not test the 160 μg dose.

Ethical approval for the study was given by the Institutional Review Boards of Noguchi Memorial Institute for Medical Research (NMIMR-IRB CPN 023/08-09) and the US Naval Medical Research Unit 3 (NAMRU3.2010.0005-IR-CONV-M (N3 1005)). All subjects provided written informed consent before their inclusion in the study. The study was registered at ClinicalTrials.gov, (Identifier: NCT01026246).

### Study site and subjects

The study was conducted at the Clinical Trial Unit of the NMIMR at University of Ghana, Legon. Healthy adults between the ages of 18 and 40 years were recruited from within and around the University of Ghana community. Written informed consent was provided by all subjects before any protocol procedures were performed. Subjects were screened per study inclusion and exclusion criteria for eligibility. Female subjects were neither pregnant nor nursing and all subjects were deemed capable of following study procedures including practicing adequate contraception and being available for the duration of the study.

### Vaccine

The study product, EBA-175 RII-NG protein formulated with Adju-Phos aluminum adjuvant, was developed under the direction of National Institute of Allergy and Infectious Diseases, Division of Microbiology and Infectious Diseases (DMID) by Leidos, Frederick, Maryland, (contract No. N01-AI-05421). The recombinant EBA-175 RII-NG protein was expressed in methylotrophic yeast, *Pichia pastoris* and supplied as a white, translucent, cloudy, non-particulate liquid suspension in a single-dose clear glass vials pre-mixed with Adju-Phos^®^. Each 2-ml vial of the vaccine contained 0.7 ml EBA-175 RII-NG at the required dose concentration, 5% sucrose, 0.5 mg/0.5 ml aluminum phosphate adjuvant and sodium phosphate buffer (10 mM sodium phosphate and 150 mM sodium chloride) with no preservatives. The vials were labeled with the concentration of EBA-175 RII-NG: 5 μg/0.5 ml dose; 20 μg/0.5 ml dose; 80 μg/0.5 ml dose and stored by refrigeration at a temperature of 2°C to 8°C. Normal saline supplied as 2 ml and 10 ml vials (The Fisher BioServices Repository, DMID, USA) stored at room temperature (between 20°C and 25°C) was used as placebo.

### Randomization and vaccination

Subjects were randomized to either vaccine or placebo group using the AdvantageEDC^SM^ data entry system (EMMES Corporation, USA). Each subject was assigned a treatment code only known to the unblinded vaccination nurse who was excluded from all post vaccination assessments in study subjects. Blinding was maintained by masking the syringe because of obvious physical differences between the vaccine (turbid liquid) and placebo (clear fluid). Cohort enrolment was staggered with a minimum 30-minute waiting period between vaccinating the first two subjects, allowing for assessment of immediate reactions of the first subject prior to vaccinating the second. The first dose was injected intramuscularly into the right or left deltoid as preferred by the subject while subsequent doses were administered in alternating arms. After the initial 2 subjects, the next 18 subjects in the group were enrolled in subgroups of 6 with a 2-day waiting period between each subgroup allowing for the assessment of reactogenicity, at least 24 hours before enrolling additional subjects. Vaccine or placebo was given at 0, 1 month and 6 months. Systemic and local reactogenicity events were observed in clinic at 30-minutes and subsequently on days 2, 7 and 14 post-vaccination. Subjects were given memory aids to record solicited (specified in the protocol) and unsolicited (not specified in the protocol) local and systemic reactogenicity information, including 12 hourly body temperature through the first 14 days after vaccination. Subjects were also assessed at 28 days, 3 months and 6 months following the third vaccine dose. In addition, information on administration of concomitant medication was also collected in the memory aid. Serious Adverse Events (SAEs) were collected throughout the study with a final study visit at Day 348 after the initial vaccination. Dosage escalation and administration of the third vaccination of a dose proceeded based on the review of safety data collected up to Day 14 after the completion of follow-up on the first two vaccinations of that cohort.

### Safety monitoring committee

The safety monitoring committee (SMC) for the trial was made up of four medical experts from: DMID, Cincinnati Children’s Hospital Medical Center, Novartis Institutes of Biomedical Research, Cambridge, Massachusetts and Baylor College of Medicine, Houston, Texas, respectively. In addition, an Independent Safety Monitor, a Professor of Medicine & Therapeutics; and Tropical Clinical Pharmacology from the University of Ghana was assigned to the study with the primary responsibility of providing independent safety monitoring in a timely fashion. The SMC convened to review the blinded safety data summaries on the first 2 doses of each cohort compiled by the EMMES Corporation in an open session. Based on their recommendations the decision was made for dosage escalation.

### Outcome Measures

#### Primary outcome: safety and reactogenicity

The primary outcome included the number of subjects spontaneously reporting any severe adverse events (Grade 3) considered associated with the vaccination at any point during the study period. Solicited local adverse reactions were graded as either mild, moderate or severe–Grades 1; 2 and 3 respectively. Pain at the injection site was categorized as Grade 1: did not interfere with activity; Grade 2: interfered with activity; Grade 3: prevented daily activity. Tenderness was graded as Grade 1: mild to touch; Grade 2: pain with movement and Grade 3: significant pain at rest; while erythema and edema measured at greatest single diameter were Grade 1: > 0–<30 mm; Grade 2: ≥ 30–<120 mm and Grade 3: ≥120 mm. Grade 1 induration measured >0–<15 mm; Grade 2: 15–30 mm and Grade 3: >30 mm. The study primary endpoints were the number of subjects experiencing: 1) Grade 3 solicited injection site reactions; 2) Grade 3 solicited systemic reactions; and 3) Grade 3 clinical laboratory values (S1 Table), within 14 days following vaccinations.

#### Secondary outcome: immunogenicity

Venous blood (35 ml) was collected from each subject at each vaccination day (Days 0, 28 and 180) and also 14 days after each vaccination (Days 14, 42 and 194) for secondary outcome (immunogenicity) analysis. The secondary outcome measures were: 1) Plasma anti-EBA-175 RII-NG IgG antibody level measured by enzyme linked immunosorbent assay (ELISA) at days 0, 14, 28, 42, 180 and 194; 2) the number of subjects experiencing a 4-fold increase in anti-EBA-175 RII-NG IgG antibody level at days 14, 28, 42, 180 and 194 relative to baseline and 3) relative growth inhibition of *P*. *falciparum* cultured *in vitro* in the presence of IgG from subjects at days 0, 42, 180 and 194.

### Antibody measurement

Immunoglobulin (Ig) G antibodies against EBA-175 RII-NG in the plasma of study subjects at days 0, 14, 28, 42, 180 and 194 were measured by ELISA as previously described [28]. Briefly, a positive plasma pool was created from samples of four Ghanaian adults with high levels of IgG to the EBA-175 RII-NG antigen. The positive pool was then standardized by running an ELISA with serial dilutions from 1:1000 to 1: 2048000 to determine the titer corresponding to an optical density (OD) of 1.0. This plasma pool was assigned 8,000 ELISA units, since a plasma dilution of 1:8,000 gave an OD of 1.0. Two-fold serial dilutions of this standardized positive pool were run in duplicate on each ELISA plate, and the standardized units of study samples were determined from curves generated by a four-parameter curve fit program (SOFTmax PRO ver.3; Molecular Devices Co., Sunnyvale, CA) for all subsequent assays. Study samples were run in triplicates at 1:500 and 1:5000 and tests were repeated if the R^2^ values for the fit was less than 0.997 and/or OD of study samples were outside the linear region of the standard curve. Responses were reported as standardized ELISA units. Commercially obtained plasma from 24 malaria-naive US volunteers were included in the ELISAs as negative control samples. The mean ELISA units and standard deviations were calculated for the negative control samples. Response of the study samples were positive if the ELISA unit was greater than the mean plus 2 standard deviations of the negative control ELISA units (i.e. 258.81 ELISA units). Thus, immunogenicity was reported using the magnitude of responses of study samples (ELISA units) as well the frequency of positive responses.

### Parasite Growth Inhibition Assay (GIA)

The *in vitro* GIA was performed as previously described [27, 29]. Briefly, protein-G purified polyclonal IgG was obtained from subjects’ plasma before initial vaccination (Day 0), 14 days after 2^nd^ vaccination (Day 42), before the 3^rd^ vaccination (Day 180) and 14 days after the 3^rd^ vaccination (Day 194). The GIA wells contained synchronized *P*. *falciparum* trophozoites and schizonts of the 3D7 strain at parasitemia of 0.3% ± 0.1%, test IgG (10 mg/ml, Nanodrop measurement) and culture media mixed at 1% hematocrit, in triplicate wells of a 96-well microtiter tissue culture plates. Control wells on each plate included wells containing: 1) uninfected red blood cells (RBCs) only; 2) infected RBCs without any IgG; 3) infected RBCs with 1.5 mM EDTA; and 4) infected RBCs with anti-AMA1-C1 rabbit IgG (positive control). After a 40-hour incubation at 37°C, parasite lactate dehydrogenase was assayed per well and the percent inhibition was calculated:

$$\text{Percent inhibition}=100-\left[\left(\frac{\left(A_{650}\;\text{of test IgG}-A_{650}\;\text{of normal RBCs}\right)}{\left(A_{650}\;\text{of infected RBCs without any IgG}-A_{650}\;\text{of normal RBCs}\right)}\right)100\right]$$

where A_650_ indicates absorbance measured at 650 nm.

### Statistical Analysis

This was a phase I study to assess adverse event (AE) rates and patterns of immune responses to ascending EBA-175 RII-NG vaccine dosages. The upper bound for a one-sided 95% confidence interval for the probability of such an event that met a pre-defined halting criteria was 0.15. The primary analysis was conducted on data collected until Day 208 (28 days following the third immunization) for each dosage cohort. The study was not statistically powered to reject secondary hypothesis of dose-related immunogenic response unless that response was very large. No formal statistical hypothesis testing of comparisons between vaccine and placebo group was done and the results presented are mainly descriptive. The control group served primarily as a bias and laboratory control. Prevalence estimates are presented with their 95% confidence intervals.

## Results

### Characteristics of study population

**Fig 1:**
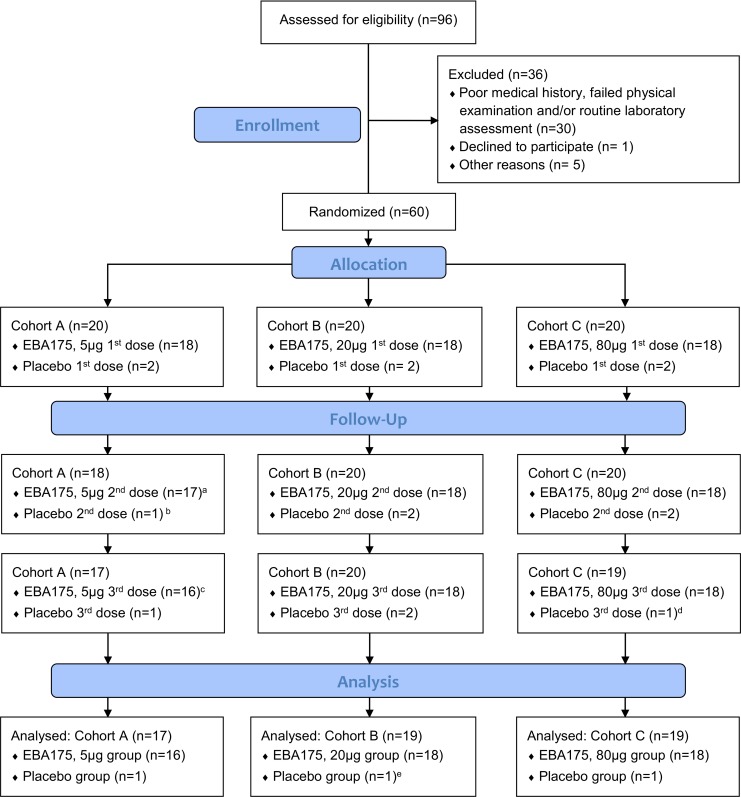
Trial profile. ^a^ Vaccination was discontinued in one subject due to blood phobia. ^b^ One subject was excluded from further vaccinations due to severe anemia. ^c^ One subject migrated out of the country without prior notification. ^d^ One subject voluntarily withdrew to relocate to another town. ^e^ One subject discontinued the study and was lost to follow up for safety evaluation after receiving all three vaccinations. All attempts to find this subject failed. Abbreviation: EBA-175 RII-NG–Erythrocyte binding antigen 175 region II non-glycosylated.

In June 2010, 96 adults were screened and 60 subjects comprising 52 males and 8 females who met the eligibility criteria were enrolled and randomized into the three cohorts (Fig 1). The overall mean age at enrolment was 24 years (range: 18–36 years) (Table 1). A total of five subjects discontinued the study at various stages of the trial for different reasons. Vaccination was discontinued in one subject after the first administration of 5 μg EBA-175 RII-NG due to blood phobia. The subject could not stand the sight of blood being drawn from his arm which made the attending physician decide to withdraw him from the study. Another subject in the placebo group was excluded from further vaccinations after the first dose due to severe anemia possibly as a result of a previous malaria episode. However, both subjects continued with safety assessment without further blood draw. One subject migrated out of the country after the second vaccination in the 5 μg group without prior notification and was lost to follow up, while another in the placebo group voluntarily withdrew after two vaccinations to relocate to another town. The fifth subject who discontinued the study was also in the placebo group and was lost to follow up for safety evaluation after receiving all three vaccinations. All attempts to find this subject failed. The final follow up for all participants was in March, 2012.

**Table 1:** Demographic and baseline characteristics by dose of study population.

	Study Group				
	Placebo (N = 6)	5 μg (N = 18)	20 μg (N = 18)	80 μg (N = 18)	Total (N = 60)
Gender: n (%)					
Male	5 (83.3)	17 (94.4)	14 (77.8)	17 (94.4)	53 (88.3)
Female	1 (16.7)	1 (5.6)	4 (22.2)	1 (5.6)	7 (11.7)
Age (Years)					
Mean (SD)	25.3 (5.54)	23.7 (3.74)	23.7 (2.76)	23.4 (3.18)	23.8 (3.47)
Min, Max	21, 36	20, 34	18, 29	18, 33	18, 36
Baseline anti-EBA-175 RII-NG IgG levels (ELISA units)					
Median	248.7	212.7	387.1	257.6	
Min, Max	96.8, 55363.1	109.8, 10297.0	110.8, 14417.0	66.2, 7021.0	
Baseline anti-EBA-175 RII-NG IgG GIA activity levels (%)					
Median	7.5	12.0	15.5	13.5	
Min, Max	-5.0, 77.0	-5.0, 36.0	-1.0, 39.0	-1.0, 48.0	

Note: Denominator for the percentages of gender is the number of subjects in the population for each dose. All study participants were Ghanaians. Abbreviations: EBA-175 RII-NG—Erythrocyte binding antigen 175 region II non-glycosylated; IgG–Immunoglobulin G antibody; ELISA–Enzyme linked immunosorbent assay; GIA–Growth inhibition assay.

The analysis population for demographic and safety data included all subjects who received at least one dose. Immunogenicity analyses exclude data points that followed a missed vaccination for subjects who were discontinued vaccination.

### Safety and reactogenicity

#### Systemic solicited adverse events

All three vaccine doses were well tolerated; no serious adverse events were reported throughout the study follow up even to the final visit on Day 348. The majority of subjects did not experience any of the solicited adverse reactions to the vaccine (Table 2). Most adverse events (AEs) observed were of either Grade 1 or Grade 2 severity and subsided during the 14 or 28-day follow up without sequelae.

**Table 2:** Adverse events by severity vaccine dose and frequency of vaccinations.

		Vaccination 1				Vaccination 2				Vaccination 3			
Adverse event	Severity	EBA17 (5 μg) N = 18	EBA175 (20 μg) N = 18	EBA175 (80 μg) N = 18	Placebo N = 6	EBA175 (5 μg) N = 17	EBA175 (20 μg) N = 18	EBA175 (80 μg) N = 18	Placebo N = 5	EBA175 (5 μg) N = 16	EBA175 (20 μg) N = 18	EBA175 (80 μg) N = 18	Placebo N = 4
**Systemic**													
Arthralgia	Any	1	0	3	1	0	3	2	0	2	3	0	0
	Grade 3	0	0	0	0	0	0	0	0	0	0	0	0
Chills	Any	2	2	0	1	0	2	1	0	1	4	1	0
	Grade 3	0	0	0	0	0	0	0	0	0	0	0	0
Fatigue	Any	1	6	4	2	3	4	1	0	4	8	1	0
	Grade 3	0	0	0	0	0	0	0	0	0	0	0	0
Fever	Any	4	2	0	1	0	0	0	0	2	2	1	0
	Grade 3	0	0	0	0	0	0	0	0	0	0	0	0
Headache	Any	8	7	5	2	6	2	2	0	4	7	4	0
	Grade 3	0	0	0	0	0	0	0	0	0	0	0	0
Malaise	Any	4	6	2	3	0	5	2	0	4	2	2	0
	Grade 3	0	0	0	0	0	0	0	0	0	0	0	0
Myalgia	Any	3	2	1	1	1	2	3	0	3	1	2	0
	Grade 3	0	0	0	0	0	0	0	0	0	0	0	0
Nausea	Any	4	1	0	1	1	2	2	0	1	1	1	0
	Grade 3	0	0	0	0	0	0	0	0	0	0	0	0
Oral temp.*	Any	2	1	0	1	1	0	0	0	1	2	1	0
	Grade 3	0	0	0	0	0	0	0	0	1	0	0	0
Vomiting	Any	3	1	0	1	0	0	0	0	0	1	0	0
	Grade 3	0	0	0	0	0	0	0	0	0	0	0	0
**Local**													
Edema	Any	1	1	0	0	0	0	0	0	2	1	0	0
	Grade 3	0	0	0	0	0	0	0	0	0	0	0	0
Erythema	Any	0	1	0	0	0	1	0	0	0	0	0	0
	Grade 3	0	0	0	0	0	0	0	0	0	0	0	0
Induration	Any	0	1	0	0	3	2	0	0	2	1	0	0
	Grade 3	0	0	0	0	0	0	0	0	0	0	0	0
Pain	Any	8	8	7	1	7	10	8	0	7	10	9	0
	Grade 3	0	0	0	0	0	0	0	0	0	0	0	0
Tenderness	Any	9	13	8	0	5	10	11	0	6	13	13	0
	Grade 3	0	0	0	0	0	0	0	0	0	0	0	0

Each subject's maximum severity is counted so that each subject appears only once for each reactogenic event. Severity described as ‘Any’ comprised Grade 1 and Grade 2 adverse events. Grade 1 (mild): did not interfere with activity; Grade 2 (moderate): interfered with activity; Grade 3 (severe): prevented daily activity.

*The recorded Grade 3 oral temperature was 40.2°C.

**Fig 2:**
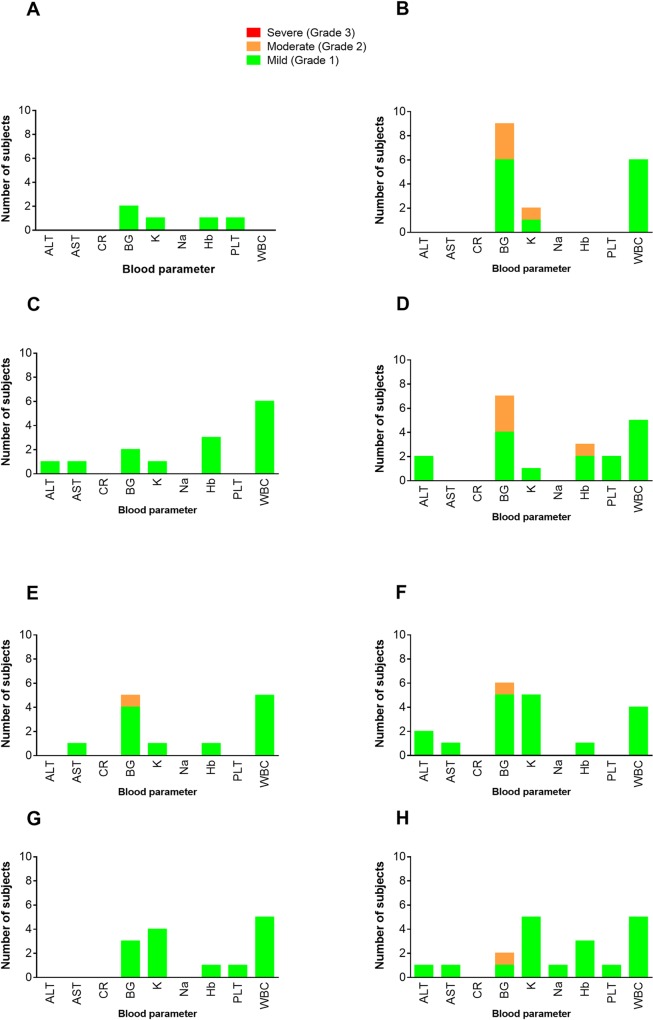
Observed laboratory adverse events for all dose escalation groups. **Panel A** is for screening prior to enrolment; **Panels B** and **C** are for vaccination 1, Day 0 and Day 14 respectively; **Panels D** and **E** are for vaccination 2, Day 0 and Day 14 respectively and **Panels F, G** and **H** are for vaccination 3, Day 0, Day 14 and Day 28 respectively. Abbreviations: ALT—Alanine Transaminase; AST—Alanine Aminotransferase; CR–Creatinine; BG–Blood glucose; K–Potassium; Na–Sodium; Hb–Hemoglobin; PLT–Platelets; WBC–White Blood Cells.

Only 1 Grade 3 AE (oral temperature of 40.2°C) was observed during the study in one subject in the 5 μg dose group on the 1^st^ day after the 3^rd^ vaccination. This was found to be due to acute *P*. *falciparum* malaria. This study subject responded to standard treatment and the excessive temperature resolved by Day 2 post vaccination. The most common systemic AE reported at all dose levels after any vaccination, was mild to moderate headache, by 36 and 11 subjects respectively (Table 2). Neither the severity nor the number of subjects experiencing headache had any clear relationship with the vaccine dose or the number of vaccinations received. Two subjects in the placebo group experienced Grade 1 headache after the first vaccination. Adverse events were generally lower among subjects receiving placebo (Table 2). Hematological and blood chemistry values were normal for at least 85% of subjects on all evaluation visits. Elevated (Grade 1) random blood glucose was the commonest biochemical AE observed in addition to one subject having low (Grade 1) hemoglobin level on the day of second vaccination (Fig 2).

#### Local solicited adverse events

The most common injection site symptoms reported were pain and tenderness, none of which was graded as severe (Table 2). Four subjects reported moderate (Grade 2) tenderness after the first dose of vaccination, seven after the second dose, and twelve after the third dose (Table 2). The remaining 65 reports of injection site tenderness across all dose levels, after any vaccination, were all Grade 1. Similarly, all reported injection site pain were Grade 1 in severity except for two subjects from 20μg and 80μg groups who experienced Grade 2 pain after the third vaccination. Although the study was not powered to detect statistically significant associations, there was no obvious relationship between the vaccine dose or vaccination frequency and the severity of the local AEs or the number subjects experiencing them (Table 2). Solicited injection site reactions were generally limited to the EBA-175 RII-NG vaccine group. With the exception of one subject in the placebo group who reported mild (Grade 1) pain after the first vaccination, no other local solicited AE was reported from the placebo group, at any vaccination (Table 2).

#### Unsolicited adverse events

A total of 209 unsolicited AEs were observed during the post vaccination follow up, mostly abnormal laboratory values of varying severity; 173 mild, 34 moderate and 2 severe. Thirty-eight mild and four moderate AEs were deemed associated with the study product, as no other reason could be ascribed. The most common associated laboratory abnormalities were leukopenia, anemia, hypoglycemia, hypokalemia and increased urine urobilinogen. Upper respiratory tract infection, headache and malaria were the most frequent unsolicited AE, unassociated with the study product. There were two severe AEs; dysmenorrhea and urinary tract infection, unassociated with the vaccine, experienced by one subject in the 20 μg dose group. The incidence of vaccine-related AEs was similar among all treatment groups.

### Immunogenicity

**Fig 3:**
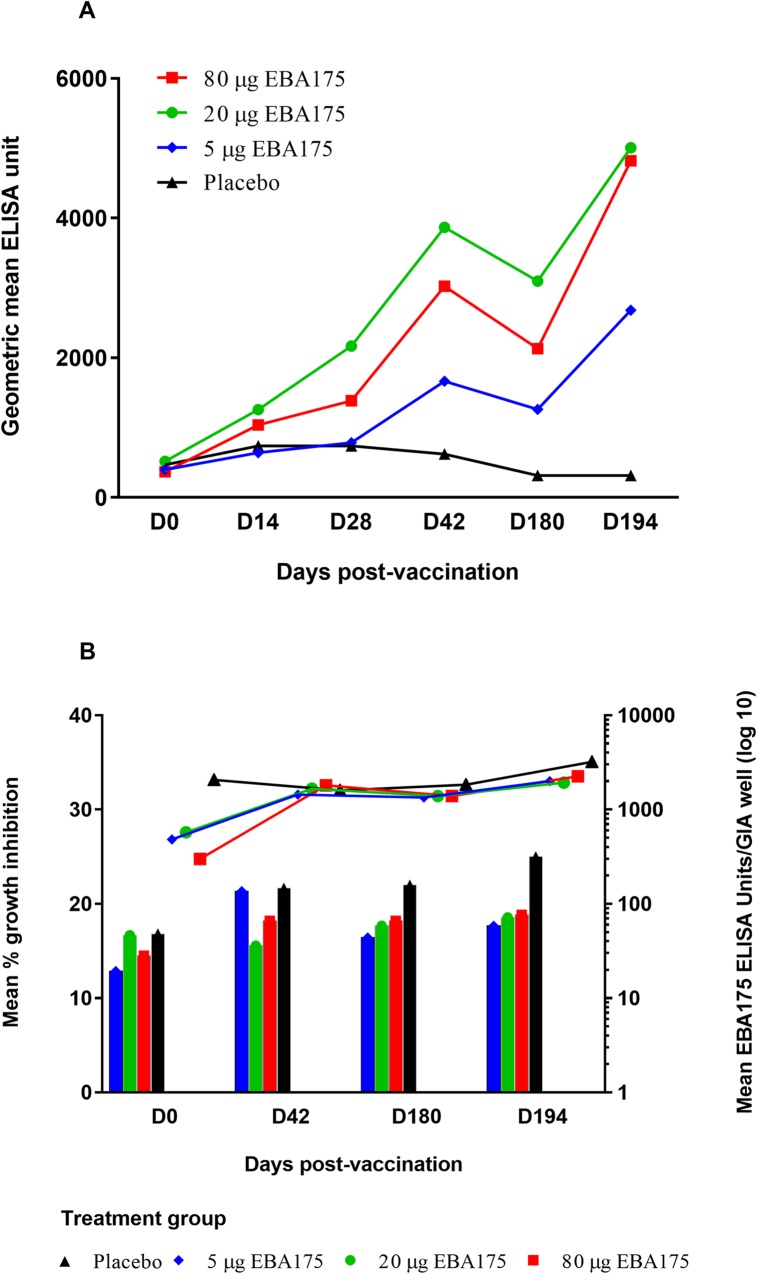
Anti-EBA-175 RII-NG IgG antibody levels (ELISA units) and growth inhibition activity against *P*. *falciparum* 3D7 parasite. **Panel A:** Geometric mean IgG antibody levels (ELISA units) to the vaccine antigen EBA-175 RII-NG measured in each treatment group on different days (D0 to D194) post vaccination. **Panel B:** The left y-axis represents mean percentage *P*. *falciparum* 3D7 parasite growth inhibition measured for each treatment group on the different days post vaccination plotted as vertical bars. The right y-axis represents mean EBA175 ELISA units (on log 10 scale) used per growth inhibition assay well for each treatment group on the different days post vaccination plotted as lines. One subject in the placebo group recorded the highest anti-EBA-175 RII-NG IgG levels at baseline which persisted throughout the study. Both the high mean EBA 175 ELISA units/GIA well and mean GIA values observed in the placebo group were largely due to this subject. One ELISA unit is the reciprocal of the dilution required to give an optical density = 1 in the standardized assay. Any data point less than the minimal detectable level was assigned as 5 ELISA units in the growth inhibition assay well in the analysis. Vaccinations occurred on Days 0, 28 and 180. Blood samples were drawn for immunogenicity prior to vaccination. Abbreviations: EBA-175 RII-NG–Erythrocyte binding antigen 175 region II non-glycosylated; IgG–Immunoglobulin G; GIA–Growth inhibition assay.

Anti-EBA-175 RII-NG ELISA was performed on plasma samples obtained on study days 0; 14; 28; 42; 180 and 194. Geometric mean anti-EBA-175 RII-NG IgG levels were low on Day 0 for all treatment groups but increased markedly in the EBA-175 RII-NG vaccine groups after the first two vaccinations. For all the EBA-175 RII-NG vaccinated groups, the geometric mean antibody levels declined after Day 42 and subsequently increased sharply after Day 180, following the third vaccination (Fig 3A). Overall, the highest antibody level was observed in the 20 μg dose group followed by the 80 μg, 5 μg and then the placebo groups (Fig 3A). One subject in the placebo group recorded the highest anti-EBA-175 RII-NG IgG level at Day 0 (55,363.12 ELISA units) (Table 1) which persisted throughout the study till day 194 (33,531.34 ELISA units).

The ELISA data were further categorized into positive or negative responses. A positive ELISA response was defined prior to analysis as more than 258.81 ELISA units. The frequency of positive ELISA response on Day 0 (pre-vaccination) ranged from 44% (8/18) in the 5 μg vaccine group to 61% (11/18) in the 20 μg group. Post vaccination, all the subjects who received 20 μg of the vaccine product showed a positive ELISA response by Day 28. All the subjects who received either 5 μg or 80 μg, showed a positive ELISA response by Day 42 and Day 194 respectively.

The proportion of subjects with a 4-fold increase in anti-EBA-175 RII-NG IgG levels from baseline increased with increasing number of vaccine doses received. The proportions of 4-fold increases was higher in the 20 μg and 80 μg vaccine groups than in the 5 μg or placebo groups at all vaccinations (Table 3). By the second vaccination (Day 28), more than half (55.6%, 95%CI = 30.8, 78.5) of subjects in the 20 μg vaccine group had a 4-fold increase in EBA-175 RII-NG antibody levels. There was a decline in the number of subjects with a 4-fold increase in anti-EBA-175 RII-NG IgG levels from baseline on Day 180 in all the vaccine groups except for the 20 μg EBA175 group (Table 3). The largest proportion (88.9%, 95%CI = 65.3, 98.6) of subjects reaching the 4-fold antibody threshold was observed in the 80 μg vaccine group at Day 194 (Table 3).

**Table 3:** Proportion of subjects with 4-fold increase from baseline in anti-EBA175 antibody response.

Study Day	Treatment Group	Number Tested	Number of Responders	95% CI for Responders
Day 14	5 μg EBA-175	18	1 (5.6)	[0.1, 27.3]
	20 μg EBA-175	18	5 (27.8)	[9.7, 53.5]
	80 μg EBA-175	18	5 (27.8)	[9.7, 53.5]
	Placebo	6	1 (16.7)	[0.4, 64.1]
Day 28	5 μg EBA-175	18	3 (16.7)	[3.6, 41.4]
	20 μg EBA-175	18	10 (55.6)	[30.8, 78.5]
	80 μg EBA-175	18	7 (38.9)	[17.3, 64.3]
	Placebo	6	1 (16.7)	[0.4, 64.1]
Day 42	5 μg EBA-175	17	9 (52.9)	[27.8, 77.0]
	20 μg EBA-175	18	13 (72.2)	[46.5, 90.3]
	80 μg EBA-175	18	13 (72.2)	[46.5, 90.3]
	Placebo	5	0 (0)	[0, 0]
Day 180	5 μg EBA-175	16	5 (31.3)	[11.0, 58.7]
	20 μg EBA-175	18	14 (77.8)	[52.4, 93.6]
	80 μg EBA-175	18	11 (61.1)	[35.7, 82.7]
	Placebo	5	0 (0)	[0, 0]
Day 194	5 μg EBA-175	16	10 (62.5)	[35.4, 84.8]
	20 μg EBA-175	18	13 (72.2)	[46.5, 90.3]
	80 μg EBA-175	18	16 (88.9)	[65.3, 98.6]
	Placebo	3	0 (0)	[0, 0]

Vaccinations occurred on Days 0, 28 and 180. Blood samples were drawn for immunogenicity prior to vaccination.

### Growth inhibition

The mean EBA-175 RII-NG ELISA units per assay well, at all time-points, were similar for the different vaccine groups. The placebo group always had higher mean EBA-175 RII-NG ELISA units per assay well compared to the vaccine groups except on Day 42 (Fig 3B). Overall, parasite growth inhibitory activity was low (range of mean percentage inhibition was 12.9% to 25.0%) and similar for the three vaccine groups on the different post-vaccinations days. The placebo group recorded the highest mean growth inhibition of 25.0% compared to any of the three vaccine groups (Fig 3B).

## Discussion

This study is the first to evaluate the safety, reactogenicity and immunogenicity of EBA-175 RII-NG vaccine adjuvanted with aluminium phosphate in a malaria exposed semi-immune adult population in an endemic country. All the three vaccine doses were found to be safe and well tolerated in this study population. There were no serious AEs throughout the study and no severe AEs associated with the vaccine at final visitation day (Day 348). The one subject who had an oral temperature of 40.2°C post vaccination 3, was found to be suffering from malaria and was successfully treated. The severe anemia reported in the subject in the placebo group who was withdrawn after the first vaccination was attributed to a previous malaria episode. The subject however, recovered fully without any sequelae and the hemoglobin measured at Day 41 after the severe anemia was 14.5g/dl. The most common vaccine site reaction was pain and tenderness, none of which was severe and the frequency was not dependent on number of vaccinations or vaccine dose. Unsolicited AEs were mostly mild and moderate and were reported by almost all of the study subjects. There were no severe laboratory abnormalities associated with the vaccine. Adverse events associated with vaccination were commonly mild to moderate laboratory abnormalities with no other known cause. There was no difference in the incidence of related AEs between treatment groups. The safety and reactogenicity results from this study are very similar and consistent with the results obtained from the Phase I study in healthy malaria naive adults conducted in the United States [27]. In the malaria naive study most AEs were moderate and the three severe AEs reported were not associated with vaccination. Pain at the injection site followed by erythema was the most prevalent symptoms reported in that study [27].

In individuals naturally exposed to malaria, antibodies against EBA-175 are usually lower compared to other blood stage antigens such as apical membrane antigen 1 (AMA1), merozoite surface protein (MSP) 1 or MSP2 [30–32]. After vaccination with the study product at any concentration, EBA-175 RII-NG antibody levels measured by ELISA and the proportion of positive responders were higher than those of the placebo group, indicating that the vaccine was immunogenic among the semi-immune Ghanaian adult population studied. However the study was not sufficiently powered to come to a conclusion about the immunogenicity of the vaccine. The levels of induced IgG antibodies after the 2^nd^ vaccination waned during the long follow-up duration of 138 days, but markedly increased again 14 days after the 3rd vaccination. The present study did not assess the IgG subclass profile of the study subjects. However, it has been shown in Mozambican infants that IgG subclass responses to EBA175 are differentially associated with *P*. *falciparum* infection outcome [33]. In the Mozambican study, a two-fold increase in IgG1 or IgG3 was significantly associated with a decrease in malaria incidence while the same level increase in IgG4 was associated with increased malaria incidence [33]. It would therefore be of interest in future trials to include the assessment of both IgG subclass profile and the dynamics of EBA-175 induced immunological memory with vaccinations since these are important parameters in malaria vaccine design [34].

The vaccine group receiving 20 μg of the test product had consistently higher EBA-175 RII-NG antibody levels compared to the 80 μg and 5 μg groups. A similar trend was observed in the previous study conducted in malaria naïve adults in the US, where the group that received 80 μg EBA-175 vaccine had higher antibodies than the 160 μg group [27]. Taken together, these findings suggest that beyond a certain vaccine dose threshold, the immunogenicity of EBA-175 RII-NG vaccine declines and the threshold may differ in malaria exposed versus non-exposed individuals. However, further investigations are necessary since sample sizes in both the previous [27] and current study were small. Such studies should also ascertain whether this phenomenon has any host immunogenetic basis since in the current study, certain individuals in the 80 μg vaccine group recorded very high anti-EBA-175 RII-NG IgG responses and the proportion of responders defined as four-fold increase over pre-vaccination levels was highest in this group compared to the 5μg and 20μg groups on day 194. The parasite growth inhibitory activity of IgG purified from plasma of those who received the vaccine were generally low and not distinctly different among the various vaccine dose groups. Interestingly, one subject in the placebo group had extremely high EBA-175 RII-NG IgG levels at Day 0 and this persisted throughout the study period. The reasons for this observation remains unclear. The case report forms indicated that this subject had mild hypokalemia after the 3^rd^ vaccination but this does not explain the persistence of high EBA-175 RII-NG IgG levels since no other subject with this AE showed such high antibody titers. At any of the measured time points, this subject had antibody levels that were at least 18 fold higher than any other subject in the placebo group and also recorded the highest GIA values in the entire study. This explains why both the mean EBA-175 ELISA units per GIA well and the mean percentage growth inhibition were often higher for the placebo group than the vaccine groups as shown in Fig 3B. The generally low growth inhibitory activity of EBA-175 RII-NG antibodies on *P*. *falciparum* growth observed in this study is consistent with findings from the previous phase I study conducted in the US in malaria naïve subjects [27]. Efficacy was not an endpoint in the current trial. However, it is noteworthy that 22.2% of vaccinated subjects had malaria during the course of the study compared to 50% of subjects who received a placebo. The low parasite growth inhibitory activity measured may partly explain the incidence of malaria in the study population since in another study, Malian children with growth inhibitory activity of <40% were shown to be at a significantly higher risk of malaria [35]. However, acquisition of functional antibodies against EBA-175 was recently shown to increase with exposure to malaria infection in Papua New Guinean children [36], suggesting that biologically active antibodies by such a vaccine may be boosted through natural infection. The functionality of antibodies elicited by EBA-175-RII-NG vaccine may also be improved by testing different adjuvant or delivery systems, as have been demonstrated for the MSP1_42_-C1 and AMA1 vaccines [37, 38].

In conclusion, the EBA-175 RII-NG vaccine was found to be safe, well tolerated and immunogenic in malaria semi-immune Ghanaian adults. The vaccine induced functional antibodies that inhibited *P*. *falciparum* growth in vitro, albeit at low levels. The study supports further development of the EBA-175 RII-NG vaccine and recommends exploration of methods to improve antibody functionality.

## Supporting Information

S1 CONSORT Checklist (DOC)

S1 Protocol (PDF)

S1 Table Laboratory adverse event grading scale. Population based reference intervals for common blood haematological and biochemical parameters; Koram *et al*., 2007 [39]. For women, results apply only if not menstruating. ** Random plasma glucose was drawn on volunteers. Elevated glucose was confirmed by repeat fasting plasma glucose. F: Female; M: Male; ALT: alanine aminotransferase; AST: aspartate aminotransferase; WBC: white blood cell; ULN: upper limit of normal
(DOC)
