# Supplementary material for: Safety and Immunogenicity of EBA-175 RII-NG Malaria Vaccine Administered Intramuscularly in Semi-Immune Adults: A Phase 1, Double-Blinded Placebo Controlled Dosage Escalation Study
Source: PLoS One. 2016 Sep 19;11(9):e0163066. doi: 10.1371/journal.pone.0163066 (PMC5028127; doi:10.1371/journal.pone.0163066)
Supplement: S1 Protocol — (PDF) [file pone.0163066.s002.pdf]

Phase 1, Double-Blinded, Placebo-Controlled Dosage-Escalation Study of the Safety and Immunogenicity of EBA-175 RII-NG Malaria Vaccine Administered Intramuscularly in Semi-immune Adults.

DMID Protocol Number: 08-0009

DMID Funding Mechanism: Contract #: HHSN266200400016C

IND Sponsor: DMID

Principal Investigator: Kwadwo A. Koram, MB, ChB, MPH&TM, PhD

Protocol Champion: Steven Rosenthal, MD, MPH

DMID Clinical Project Manager: Walter Jones, RN, MPH

DMID Medical Monitor: Gino Girardi, MD

DMID Regulatory Affairs Specialist: Blossom Smith, M.S.

Version Number: 4.0

Day Month Year

12 August, 2010

Statement of Compliance

This trial will be conducted in compliance with the protocol, International Conference on Harmonisation (ICH) E6: Good Clinical Practice (GCP): Consolidated Guideline and the applicable regulatory requirements. The applicable regulations and requirements include:

- U.S. Code of Federal Regulations (CFR) applicable to clinical studies (45 CFR 46 and 21 CFR including parts 50 and 56 concerning informed consent and institutional review board (IRB) regulations, if under investigational new drug (IND), 21 CFR 312).
- ICH E6; 62 Federal Register 25691 (1997).

All key personnel (all individuals responsible for the design and conduct of this study) have completed human subjects protection training.

RESTRICTED ACCESS

This document is a restricted access communication. Receipt of this document constitutes agreement by the recipient that no unpublished information herein shall be published or disclosed without prior written approval, except that this document may be disclosed to the appropriate IRB under the condition that they keep it confidential.

Signature Page

The signature below constitutes the approval of this protocol and the attachments, and provides the necessary assurances that this trial will be conducted according to all stipulations of the protocol, including all statements regarding confidentiality, and according to local legal and regulatory requirements and applicable US federal regulations and ICH guidelines.

Principal Investigator:

Signed: ______________________________ Date: ______________

Kwadwo A. Koram, MB, ChB, MPH&TM, PhD

Table of Contents

Statement of Compliance i
Signature Page ii
List of Abbreviations vi
Protocol Summary 1

Primary: 3
1 Key Roles 4
2 Background Information and Scientific Rationale 7
2.1 Background Information 7
2.1.1 Safety and Immunogenicity in Mice 10
2.1.2 Comparative Immunogenicity Study of Nonadjuvanted EBA-175 Versus Three Different Adjuvanted EBA-175 Preparations in Male BALB/c Mice 10
2.1.3 Study of the Immunogenicity of EBA-175 in Mice When Formulated With Alhydrogel or Adju-Phos at pH6.0 and pH7.2 12
2.1.4 EBA-175 Dose Range Finding for Intramuscular Potency in BALB/c Mice of the Immunogenicity of EBA-175 When Formulated With Adju-Phos at pH7.2 12
2.1.5 EBA-175 Malaria Vaccine in Adju-Phos: 99-day Repeat Intramuscular Dose Toxicity in the New Zealand White Rabbit 13
2.2 Rationale 15
2.3 Potential Risks and Benefits 15
2.3.1 Potential Risks 15
2.3.2 Known Potential Benefits 16
2.3.3 Provision of Study-Related Care 16
3 Objectives 17
3.1 Study Objectives 17
3.2 Study Outcome Measures 17
3.2.1 Primary Outcome Measures 17
3.2.2 Secondary Outcome Measures 17
4 Study Design 19
5 Study Enrollment and Withdrawal 20
5.1 Subject Inclusion Criteria 21
5.2 Subject Exclusion Criteria 21
5.3 Treatment Assignment Procedures 23
5.3.1 Randomization Procedures 23
5.3.2 Masking Procedures 23
5.3.3 Reasons for Withdrawal 24
5.3.4 Handling of Subjects Who Discontinue Vaccination 24
5.3.5 Termination of Study 24
6 Study Intervention/Investigational Product 25

6.1 Study Product Description 25
6.1.1 Acquisition 25
6.1.2 Formulation, Packaging, and Labeling 25
6.1.3 Product Storage and Stability 25
6.2 Dosage, Preparation and Administration of Study Intervention/Investigational Product 26
6.3 Modification of Study Intervention/Investigational Product for a Participant 26
6.4 Accountability Procedures for the Study Intervention/Investigational Product(s) ...26
6.5 Concomitant Medications/Treatments 26
7 Study Schedule 28
7.1 Screening 28
7.2 Enrollment/Baseline 28
7.3 Follow-Up 28
7.4 Final Study Visit 28
7.5 Early Termination Visit 29
7.6 Unscheduled Visit 29
8 Study Procedures/Evaluations 30
8.1 Clinical Evaluations 30
8.1.1 Screening Visit Day -28 to -1, Visit 1 30
8.1.2 Day 0, Visit 2 30
8.1.3 Day 2 (± 1), Visit 3 31
8.1.4 Day 7 (± 2), Visit 4 32
8.1.5 Day 14 (± 3), Visit 5 32
8.1.6 Day 28 (± 3 days), Visit 6 32
8.1.7 Day 30 (± 1), Visit 7 33
8.1.8 Day 35 (± 2), Visit 8 34
8.1.9 Day 42 (± 3), Visit 9 34
8.1.10 Day 180 (± 14), Visit 10 35
8.1.11 Day 182 (± 1), Visit 11 35
8.1.12 Day 187 (± 2), Visit 12 36
8.1.13 Day 194 (± 3), Visit 13 36
8.1.14 Day 208 (± 7) Visit 14 37
8.1.15 (Day 258 ± 21) Visit 15 37
8.1.16 Day 348 (± 21) Visit 16 37
8.2 Laboratory Evaluations 37
8.2.1 Clinical Laboratory Evaluations 37
8.2.2 Special Assays or Procedures 38
9 Assessment of Safety 39
9.1 Specification of Safety Parameters 39
9.2 Methods and Timing for Assessing, Recording, and Analyzing Safety Parameters 39
9.2.1 Adverse Events 39
9.2.2 Reactogenicity 40
9.2.3 Serious Adverse Event 41
9.2.4 Procedures to be Followed in the Event of Abnormal Laboratory Test Values or Abnormal Clinical Findings 42

9.3 Reporting Procedures.....42
9.3.1 Serious Adverse Event Detection and Reporting.....42
9.3.2 Regulatory Reporting for Studies Conducted Under DMID-Sponsored IND.....43
9.3.3 Reporting of Pregnancy.....44
9.4 Type and Duration of Follow-Up of Subjects after Adverse Events.....44
9.5 Halting Rules.....44
9.6 Safety Oversight.....45
10 Clinical Monitoring Structure.....47
10.1 Site Monitoring Plan.....47
11 Statistical Considerations.....48
11.1 Study Overview and Design.....48
11.2 Sample Size Considerations.....48
11.3 Final Analysis Plan.....49
12 source documents and Access to Source Data/Documents.....50
13 Quality Control and Quality Assurance.....51
14 Ethics/Protection of Human Subjects.....52
14.1 Ethical Standard.....52
14.2 Institutional Review Board.....52
14.3 Informed Consent Process.....52
14.4 Exclusion of Women, Minorities, and Children (Special Populations).....53
14.5 Volunteer Recompense.....53
14.6 Subject Confidentiality.....53
14.7 Study Discontinuation.....54
15 Data Handling and Record Keeping.....55
15.1 Data Management Responsibilities.....55
15.2 Data Capture Methods.....55
15.3 Types of Data.....56
15.4 Data Requirements.....56
15.5 Study Records Retention.....56
15.6 Protocol Deviations.....56
16 Publication Policy.....58
17 Literature References.....59

APPENDICES

A: Schedule of Procedures

B: Laboratory Adverse Event Grading Scale

C: Reactogenicity Grading Scale

List of Abbreviations

AE	Adverse Event
ALT	Alanine Aminotransferase
AST	Aspartate Aminotransferase
β-HCG	Beta-Human Chorionic Gonadotropin
CBC	Complete Blood Count
CFA	Complete Freund's Adjuvant
CFR	Code of Federal Regulations
CRF	Case Report Form
DCC	Data Coordinating Center
DMID	Division of Microbiology and Infectious Diseases, NIAID, NIH
EBA-175	175 KDa-Erythrocyte Binding Antigen
EBA-175 RII	Erythrocyte-Binding Antigen 175 kDa Region II
EBA-175 RII-NG	Erythrocyte-Binding Antigen 175 kDa Region II-Nonglycosylated
eCRF	Electronic Case Report Form
EDTA	Ethylenediaminetetraacetic Acid
ELISA	Enzyme-Linked Immunosorbent Assay
GCP	Good Clinical Practice
HBsAg	Hepatitis B Surface Antigen
HCV	Hepatitis C Virus
Hgb	Hemoglobin
HIV	Human Immunodeficiency Virus
ICH	International Conference on Harmonisation
IEC	Institutional Ethics Committee
ICFA	Incomplete Freund's Adjuvant
ICMJE	International Committee of Medical Journal Editors
IM	Intramuscular
IND	Investigational New Drug
IP	Intraperitoneal
IRB	Institutional Review Board

MedDRA®	Medical Dictionary for Regulatory Activities
N	Number (typically refers to subjects)
NaCl	Sodium Chloride
NIAID	National Institute of Allergy and Infectious Diseases, NIH
NIH	National Institutes of Health
NMIMR	Noguchi Memorial Institute for Medical Research
PE	Physical Examination
Pf	*Plasmodium falciparum*
PI	Principal Investigator
RBC	Red Blood Cell
SAE	Serious Adverse Event
SAIC	Science Applications International Corporation
SC	Subcutaneous
SDCC	Statistical and Data Coordinating Center
SDW	Source Document Workbook
SMC	Safety Monitoring Committee
ULN	Upper Limit of Normal
WBC	White Blood Cell

Protocol Summary

Title: Phase 1, Double-Blinded, Placebo-Controlled, Dosage-Escalation Study of the Safety and Immunogenicity of EBA-175 RII-NG Malaria Vaccine Administered Intramuscularly in Semi-immune Adults

Phase: I

Population: 60 malaria semi-immune healthy subjects between the ages of 18 and 40 years, males and females, recruited from Accra, Ghana

Site: Noguchi Memorial Institute for Medical Research

Study Duration: Approximately 24 months

Description of Agent or Intervention:

The vaccine is a recombinant *Plasmodium falciparum (Pf)* erythrocyte-binding antigen 175 kDa Region II-nonglycosylated (EBA-175 RII-NG) adsorbed to aluminum phosphate adjuvant.

Objectives:

Primary: To assess the safety and reactogenicity (tolerability) of ascending dosages of EBA-175 RII-NG vaccine among healthy subjects given in 3 intramuscular doses at 0, 1 and 6 months.

Secondary: To evaluate the immunogenicity of the EBA-175 RII-NG vaccine by measuring anti-EBA-175 RII-NG antibodies using enzyme-linked immunosorbent assay (ELISA), inhibition of *P. falciparum* growth *in vitro*, and inhibition of binding of EBA-175 RII-NG to red blood cells (RBCs)

Description of Study Design:

Subjects will be randomized to receive 3 doses of the vaccine or saline placebo by the intramuscular route in a 9:1 ratio at 0, 1 and 6 months. The safety and immunogenicity of ascending dosages of the vaccine will be assessed. Eighteen subjects will receive vaccine at each of the following dosage levels: 5, 20, and 80 µg. Two subjects will receive placebo for each dosage level. Dosage escalation will proceed only after review of the 2-week safety data of the 2 initial doses of the prior dosage level.

Group	Vaccine Formulation	Number of Vaccine/Placebo
A	5 µg EBA-175 + 500 µg aluminum adjuvant	18/2
B	20 µg EBA-175 + 500 µg aluminum adjuvant	18/2
C	80 µg EBA-175 + 500 µg aluminum adjuvant	18/2
Total		54/6

Outcome Measures:

Primary:

- The number of subjects experiencing severe (Grade 3) solicited injection site reactions within 14 days following vaccination.
- The number of subjects experiencing severe solicited systemic reactions (Grade 3) within 14 days following vaccination.
- The number of subjects experiencing severe (Grade 3) clinical laboratory values within 14 days following vaccination.
- The number of subjects spontaneously reporting adverse events considered associated with the vaccination that are severe (Grade 3) at any point during the study period.
- Serious adverse events considered associated with the vaccination reported at any point during the study period.

Secondary:

- Anti-EBA-175 RII-NG antibody level by ELISA at days 0, 14, 28, 42, 180 and 194..
- The number of subjects experiencing a 4-fold increase in Anti-EBA-175 RII-NG antibody level (ELISA) at days 14, 28, 42, 180 and 194 relative to baseline.
- Relative growth inhibition of *Pf* in human RBCs cultured *in vitro* in the presence of serum from immunized individuals at days 0, 14, 28, 42, 180 and 194.

1 KEY ROLES

Individuals:

**Principal Investigator:**	Kwadwo A. Koram, MB, ChB. PhD<br>Noguchi Memorial Institute for Medical Research<br>University of Ghana<br>Legon
**Clinical Project Manager:**	Walter Jones, RN, MPH<br>Parasitology & International Programs Branch<br>DMID, NIAID, NIH<br>6610 Rockledge Drive, Room 5105, MSC 6604<br>Bethesda MD 20892
**Protocol Champion:**	Steven Rosenthal, MD<br>Parasitology & International Programs Branch<br>DMID, NIAID, NIH<br>6610 Rockledge Drive, Room 5105, MSC 6604<br>Bethesda MD 20892
**DMID Medical Monitor:**	Gino Girardi, MD<br>DMID/NIAID<br>Office of Clinical Research Affairs<br>6610 Rockledge Drive<br>Room 4506<br>Bethesda MD 20892-6603
**Independent Safety Monitor:**	David Ofori-Adjei, MB, ChB, MRCP

Coinvestigators:

Josephine C. Ocran, MD, MPH

Daniel Dodoo, PhD

Ben Gyan, PhD

Susan Adu-Amankwah, MPH

Francis K. Nkrumah, MD, MPH

LCDR Karl Kronman, MD, MPH, USN

Officer in Charge, Ghana Detachment

Naval Medical Research Unit No. 3

American Embassy, Accra
Mobile: +233-24-4333027
Office: +233-21-741649
karl.kronmann@med.navy.mil

Laboratory Support:

Carole A. Long, PhD

Laboratory of Malaria and Vector Research
NIAID/NIH
12735 Twinbrook Parkway, Rm. 3W- 13
Rockville MD, 20854

Institutions:

Noguchi Memorial Institute for Medical Research

University of Ghana

P. O. Box LG 581

Legon, Accra

Ghana

The EMMES Corporation

(NIH contractor for data management and statistical support)

401 N. Washington St., Suite 700

Rockville, MD 20850

Tel: 301-251-1161, Fax: 301-251-1355

Email: malaria@emmes.com

Naval Medical Research Unit No. 3

NAMRU-3
PSC 452 Box 5000
FPO AE 09835-9998
3A Imtidad Ramses Street
Adjacent to Abbassia Fever Hospital
Abbassia, Cairo, Egypt 11517

2 BACKGROUND INFORMATION AND SCIENTIFIC RATIONALE

2.1 Background Information

Malaria accounts for 500 million febrile illnesses and more than a million deaths annually. Most of the deaths occur in children under the age of 5 years old and pregnant women. The disease burden is heaviest in economically developing countries where it is estimated that up to 5% of the gross domestic product of sub-Saharan countries is consumed by the direct and indirect health costs of malaria.

Malaria is caused by four different species of *Plasmodium*: *vivax, malariae, falciparum* and *ovale.* The parasite is transmitted to humans through the bites of infected female *Anopheles* mosquitoes. Mosquitoes release the malaria sporozoites when they feed, and the sporozoites are carried to the liver. The sporozoites invade liver cells and multiply rapidly for 4 to 5 days. Tens of thousands of asexual stage merozoites are released from each infected liver cell. Merozoites then infect and multiply in erythrocytes (red blood cells, [RBCs]), resulting in hemolysis. Periodic paroxysms of chills and fever coincide with the synchronized release of parasites from the RBCs. Every few days the merozoites multiply 10-fold and burst out to infect other RBCs. The cyclic and massive increase in parasite burden give rise to the clinical disease we know as malaria. During the erythrocyte phase, some parasites switch to the sexual form of male and female gametocyte. The gametocytes are taken up by the *Anopheles* mosquito. In the mosquito midgut, male and female gametes cross fertilize to complete the life cycle of the parasite.

Plasmodium falciparum (*Pf*) and *Plasmodium vivax* cause the highest disease burden, and vaccine development has been targeted to prevent disease related to these two organisms. *Plasmodium falciparum* deserves special attention in terms of vaccine development because it causes the more severe forms of the illness and the highest mortality rates. In general, malaria causes morbidity and mortality in 2 broadly defined risk groups: 1) Individuals living in endemic areas who are at risk of recurrent infections eventually develop immunity to clinical disease caused by malaria. However, children in these areas are at the highest risk of mortality due to severe anemia and/or coma, and primigravida women due to placental sequestration;
2) malaria-naïve travelers and migrant populations crossing from malaria-free to malaria-

endemic areas. These individuals are at risk of severe disease due to acquiring their first malaria infection.

Accordingly, malaria vaccine development has thus far been aimed at developing at least 2 broad categories of vaccines: disease-modifying and infection-prevention. Disease-modifying vaccines are designed to reduce the severity of infection and prevent mortality without necessarily eradicating the infection. These vaccines are mostly targeted at the erythrocytic stages of the parasite. Infection-prevention vaccines are designed to prevent the malaria infection and are usually targeted at the pre-erythrocytic stages of the parasite lifecycle.

Malaria vaccine development has been hampered by multiple hurdles:

- Researchers have not identified an immune response that strongly predicts protection from infection or disease.
- The parasite expresses different antigens in different stages of the infection. Hence, an erythrocytic vaccine is not likely to be protective against liver infection, and the reverse is likely true.
- Many parasite proteins are highly polymorphic, which suggests that, if these proteins are to be used in a vaccine, protein sequences from various *Pf* strains should be used in combination.
- The human host exhibits a heterogeneity in the immune response to malaria infection. Immune responses and thus clinical outcomes depend on certain genetic variables including the type of human leukocyte antigen (HLA) and tumor necrosis factor-α polymorphisms.

One way to circumvent these hurdles is to develop a multicomponent vaccine targeted at the erythrocytic and the sporozoite stages. An alternate route would be to design a vaccine that generates antibodies against blood-stage parasites to prevent severe malaria-related disease and mortality. Such a vaccine should use antigen(s) that are recognized by the immune system, produce a protective immune response in animal models and have limited genetic variability. One *Pf* protein that may meet the aforementioned criteria is the 175 KDa-erythrocyte binding antigen (EBA-175).

EBA-175 is a parasite ligand that binds to its receptor glycophorin A on the surface of the erythrocyte. Using truncated portions of EBA-175 expressed on COS-7 cells, Sim, et al,

identified RII as the region of EBA-175 that specifically binds to the sialic acid moieties of glycophorin A.[1]

Sequence analysis of Region II (RII), a 616 amino acid fragment, shows complete conservation of 27 cysteine residues across more than 30 laboratory strains as well as wild isolates, indicating that the tertiary structure of RII is probably required for function. At the amino acid level, there is also strict conservation of sequences between cysteines with the exception of 5 to 13 specific residues scattered throughout RII.[2] By targeted surface expression of RII on the surface of COS-7 cells, these few changes limited to specific residues did not affect the binding function of RII. Furthermore, the sialic acid-binding specificity was conserved since none of the COS-7 cell transfectants bound to neuraminidase-treated erythrocytes.

Region II gene sequences are highly conserved. There is no evidence of gene deletion. The gene encoding EBA-175 is present in all isolates studied to date. Erythrocyte-binding antigen 175 kDa Region II (EBA-175 RII) is expressed at equivalent levels in previous studies of the following parasite strains: Camp, FCR3, 7G8 strains, and HB3, Dd2, etc. Antibodies against RII block erythrocyte invasion of sialic acid-dependent and sialic acid-independent (alternative invasive pathway) parasite strains *in vitro*.

Jones, *et al*, immunized *Aotus* monkeys with 4 doses of Pf EBA-175 RII vaccine as plasmid DNA or recombinant protein in adjuvant or as 3 doses of DNA and 1 dose of protein. Four weeks after the last immunization, the animals were challenged with 10^4 *Pf*-parasitized erythrocytes. Peak levels of parasitemia were lower in the 16 monkeys that received DNA or protein vaccine than in the 16 controls (geometric mean: 194 178 and 410 110 parasites/µL, respectively p=0.013).[3]

The National Institute of Allergy and Infectious Diseases (NIAID), Division of Microbiology and Infectious Diseases (DMID), National Institutes of Health (NIH) directed Science Applications International Corporation (SAIC) under the Malaria Vaccine Production and Support Services Program (prime contract No. N01-AI-05421) to manage process development, scale-up manufacturing, formulation, and preclinical testing in anticipation of the initiation of clinical trials by DMID of EBA-175 RII NG as a candidate malaria vaccine. Investigators at Protein Potential developed the EBA-175 RII-NG malaria vaccine using the native EBA-175 RII gene expressed in baculovirus. Due to limited productivity of baculovirus expression, the baculovirus recombinant protein vaccine was replaced by the expression of RII in the methylotrophic yeast *Pichia pastoris.* Furthermore, the *P pastoris* recombinant protein expression system allows for

the secretion of cysteine-rich proteins without the need for refolding strategies. The vaccine is mixed with Adju-Phos®, an aluminum phosphate adjuvant.

2.1.1 Safety and Immunogenicity in Mice

Male BALB/cAnNHsd mice were used to determine the potential immunogenicity of EBA-175 RII-NG. Mice (N=28) were administered 3 doses of 100 µg/animal of EBA-175 RII-NG formulated with complete Freund's adjuvant (CFA) or incomplete Freund's adjuvant (ICFA) or adjuvant control (N=27) by subcutaneous (SC) injection during a 42-day study period, on Days 1, 15, and 29 or 33. Serum (5 µL) was collected from each mouse on Days 14 and 29 and pooled. Anti-RII-NG titers as well as specific blocking titers against the native EBA-175 binding to human RBCs were determined from the pooled sera. RII antibody titers (enzyme-linked immunosorbent assay [ELISA] titer) were boosted approximately 10-fold. The specific native EBA-175 blocking titer also increased from a 1:100 to 1:500 range to 1:1000 to 1:5000 range. These data indicate that the specific blocking antibody titer increases proportionally to the RII antibody titer. No test article-related changes in mortality or clinical signs of toxicity were observed. Subcutaneous exposure of male BALB/c mice to EBA-175 RII-NG did not produce significant mortality or clinical signs of toxicity.

2.1.2 Comparative Immunogenicity Study of Nonadjuvanted EBA-175 Versus Three Different Adjuvanted EBA-175 Preparations in Male BALB/c Mice

Male BALB/c mice were used to determine the immunogenicity and toxicity of *P pastoris*-derived EBA-175 RII-NG formulated in various adjuvants when 2 doses were administered by SC injection on Days 1 and 15 of a 28-day study period. Male BALB/c mice (10 per group, 11 groups) received SC injections (100 µg total dose) of EBA-175 RII-NG formulated in different control buffers and/or adjuvants or appropriate controls as designated in Table 1.

Table 1: Test and Control Articles

Group Number	Test Article	Antigen Dose (μg)
1	Control Buffer #1[a]	0.0
2	EBA-175 alone	10.0
3	EBA-175 alone	30.0
4	EBA-175 in CFA/ICFA[b] in Control Buffer #2[c]	10.0
5	EBA-175 in CFA/ICFA	10.0
6	EBA-175/Alhydrogel®	10.0
7	EBA-175/Alhydrogel	30.0
8	Alhydrogel Control	0.0
9	EBA-175/Adju-Phos®	10.0
10	EBA-175/Adju-Phos	30.0
11	Adju-Phos Control	0.0

[a] Control Buffer 1 (EBA-175 RII-NG with 5% sucrose in 10 mM sodium phosphate buffer at a pH of 6.0) was used as the test article in all groups except 1 and 4.

[b] CFA/ICFA = Complete Freund's adjuvant/incomplete Freud's adjuvant.

[c] Control Buffer #2 (EBA-175 RII-NG with 5% sucrose in 10 mM sodium phosphate buffer at a pH of 7.2) was used as the test article in Group 4 only.

Parameters evaluated during this study period included mortality and clinical signs of toxicity, physical and cageside examinations, Draize observations, body weight, body weight changes, food consumption, and immunogenicity. Adding adjuvant induced measurable antibody titers. Without adjuvant, even dosages as high as 30 μg failed to induce an immune response. The group that received the protein with Freund's adjuvants had higher antibody titers at all time points than did the aluminum hydroxide adjuvant groups; however, the results did not reach the level of statistical significance except after the first dose using untransformed data. The group that received the protein with Alhydrogel® had higher antibody titers at all time points than did the Adju-Phos adjuvant groups; however, the results were not statistically significant. Treatment with 10 or 30 μg of EBA-175 alone or in combination with CFA/ICFA, Alhydrogel, or Adju-Phos caused no mortality and had no effect on clinical or cage-side observations. Of all the adjuvants, the animals dosed with CFA/ICFA had the most serious findings with an increased time to recovery. For the proposed study, Adju-Phos was chosen because of the extensive prior

experience with Adju-Phos in other vaccines, antigen stability assessment is easier to perform with Adju-Phos, and for future development of multi-antigen vaccines.

2.1.3 Study of the Immunogenicity of EBA-175 in Mice When Formulated With Alhydrogel or Adju-Phos at pH6.0 and pH7.2

The objectives of this study were (1) to determine if the pH of the formulation affects the immunogenicity of 2 doses of adjuvanted EBA-175 RII-NG administered by SC (1 or 10 µg antigen) or intraperitoneal (IP) injection (0.1 µg) on Days 1 and 15 of a 28-day study; (2) to determine the comparative immunogenicity of EBA-175 RII-NG adjuvanted with Alhydrogel versus Adju-Phos at pH 7.2; (3) to determine if very low doses of EBA-175 RII-NG are immunogenic in male mice when administered IP; and (4) to collect additional safety data on aluminum-adjuvanted EBA-175 RII-NG. Parameters evaluated during this study period included mortality and clinical signs of toxicity, physical and cage-side examinations, Draize observations, body weight, body weight changes, food consumption, and immunogenicity. All antigen-receiving groups produced a high antibody titer. Titers were generally higher for Alhydrogel when the formulation was made in buffer at pH 6.0; whereas titers were generally higher for Adju-Phos when the formulation was made in buffer at pH 7.2. The IP route of administration could be used as an alternative to the SC route for potency testing.

2.1.4 EBA-175 Dose Range Finding for Intramuscular Potency in BALB/c Mice of the Immunogenicity of EBA-175 When Formulated With Adju-Phos at pH7.2

The objective of this study was to determine the dose response for immunogenicity of two doses of the EBA-175 protein when formulated with Adju-Phos and administered by the intramuscular (IM) route on Days 1 and 15 of a 29-day study. Nine groups of male BALB/c mice (10 per group) received either 100 µL containing EBA-175 placebo (Group 6); or 10, 3, 1, 0.3, or 0.1 mcg of EBA-175 RII-NG by IM route (Groups 1-5); or 100 µL containing 3, 1, or 0.3 mcg EBA-175 RII-NG by the SC route (Groups 7-9). All mice were euthanized in accordance with standard operating procedures following the terminal bleed on Day 29. Parameters evaluated during this study period included mortality and clinical signs of toxicity, physical and cage-side examinations, Draize observations, body weight, body weight changes, food consumption, and immunogenicity. Results of this study demonstrated that antibody responses elicited by IM administration were comparable to antibody responses elicited by the SC route. In all cases, the mean antibody titers were greater after IM than after SC administration. However, the

differences between the 2 groups reached the level of statistical significance (*p*=0.02, Student's *t*-test, 2-tailed) only for the 0.3 µg dose group. No test article-related mortality or clinical signs of toxicity, physical and cage side examinations, body weights (including changes), or food consumption were observed.

2.1.5 EBA-175 Malaria Vaccine in Adju-Phos: 99-day Repeat Intramuscular Dose Toxicity in the New Zealand White Rabbit

The objective of this 99-day, good laboratory practice-conducted study was to determine the safety and potential toxicity of four doses of EBA-175 RII-NG drug product formulated with Adju-Phos and cGMP-manufactured by Hollister-Stier. The test article was EBA-175 RII-NG formulated in 5% sucrose in 10 mM sterile sodium phosphate buffer at a pH of 7.2 and 150 mM sodium chloride (NaCl) containing 0.5 mg aluminum as Adju-Phos adjuvant per 0.5 mL. Only the 2 highest proposed clinical dosages were tested (80 µg [Hollister-Stier Lot 6750] and 160 µg [Hollister-Stier Lot 6751]). The control article (adjuvant) was 5% sucrose in 10 mM sterile sodium phosphate buffer at a pH of 7.2 and 150 mM NaCl containing 0.5 mg per 0.5 mL of Adju-Phos adjuvant (Hollister-Stier Lot 6746). New Zealand White rabbits (12 per sex per group for a total of 72 rabbits among 3 groups), received IM doses (either 80 or 160 µg/dose split between left and right thigh muscle) of EBA-175 RII-NG in adjuvant (Group 2 or 3) or GMP placebo (adjuvant) control (Group 1) on Days 1, 29, 57, and 85. Cageside observations included observation for mortality, moribundity, general health and signs of toxicity. Clinical observations (made prior to each dose, weekly, and prior to necropsy) included evaluation of skin and fur characteristics, eye and mucous membranes, respiratory, circulatory, autonomic and central nervous systems, and somatomotor and behavior patterns. Following the last blood collection, animals were euthanized by sodium pentobarbital injection and exsanguinated. Gross necropsies, tissue collection, histopathology, organ weights, and bone marrow collection were performed. Any animals found dead prior to scheduled necropsy were only to have gross necropsies and tissue preservation performed. All animals survived to scheduled termination. No test article-related mortality or clinical signs of toxicity were observed. Study Days 88 and 99 combined grouping ELISA titer results, presented as geometric mean (standard deviation) for the 0, 80, 160 µg/dose groups, were 7 (292), 1,127,463 (2,580,801), and 458,872 (524,972), respectively.

2.1.6. Phase 1, Double-Blinded, Placebo-Controlled Dosage-Escalation Study Of the Safety and Immunogenicity of EBA-175 RII-NG Malaria Vaccine Adminstered Intramasularly.

The primary objective of this study was to assess the safety and tolerability of ascending dosages of EBA-175RII-NG vaccine in healthy, malaria naïve subjects. The secondary objective was to evaluate immunogenicity of the vaccine. Subjects were healthy non-immune adults between the ages of 18 and 40 years, randomized to receive 3 doses of vaccine or saline placebo by intramuscular route at 4 dose levels; eighteen subjects received vaccine at each dose level (5, 20, 80 and 160µg) and two subjects received placebo for each dose level. The criteria for the evaluation of safety were the frequency and severity of injection-site and systemic reactions to vaccination as well as all other adverse events and clinical laboratory abnormalities. This study was exploratory and results presented were descriptive, with no formal comparisons between vaccine and placebo groups. (Systemic and local reactogenicity symptoms were collected for 15 days following each vaccination. On the whole, following any vaccination, 3 (4%) experienced severe systemic reactions and 27 subjects (34%) experienced moderate systemic reactions. None of the 3 severe events was associated with vaccination, and all resolved without sequelae. The reactions were gastroenteritis, headache (related to burning incense), and abdominal cramps. Moderate symptoms were evenly distributed across all dose groups. Headache was the most prevalent symptom reported across all treatment groups, fatigue and malaise were the next prevalent reported symptoms. Two subjects experienced severe local reactions after the third vaccination (one in the 80µg group and the other in the 160 µg group), both experienced severe erythema and induration. Nine subjects (13%) experienced moderate local reactions following vaccination. Pain at the injection site was the most prevalent local reaction for all the treatment groups; the second-most prevalent was erythema. In total, 192 non-serious unsolicited adverse events were reported among 65 of the 80 subjects (81.3%) enrolled. Of the 192 events, 36 (18.8%, experienced by 25 subjects) were considered to be associated with the vaccination. None of the associated reported events was severe and 5 (13.9%) of the events were of moderate severity. For chemistry, two subjects experienced severe AST results, three experienced severe glucose results and two experienced severe potassium results. There were four moderate glucose results, four moderate potassium results and one each moderate AST and ALT results. No severe hematology results were reported; three subjects had moderate hematology results. ELISA titers for antibody responses peaked at Day 194, baseline titers were small relative to the Day 194 titers and the lowest 8-fold response rate at Day 194 was 83.3% in the highest dose group.

2.2 Rationale

These studies provided the preliminary data that were used in choosing the dose and route of test article administration. We propose to conduct a Phase-I dosage-escalating study to assess the safety and immunogenicity of 3 different dosages of EBA-175 RII-NG adjuvanted with Adju-Phos: 5 µg, 20 µg, and 80 µg, given in 3 doses at 0, 1, and 6 months by IM injection to healthy young adults in a malaria endemic area (semi-immune adults). One dose of vaccine will be given at each time point. We hypothesize that the vaccine is safe at the proposed dosages. The rationale for not testing a dosage of 160 ug is that there does not appear to be a significant difference in immunogenicity between the 80 ug dose cohort vs. the 160 ug dose cohort in the completed Phase 1 trial in the U.S.

2.3 Potential Risks and Benefits

2.3.1 Potential Risks

EBA-175 RII-NG has never been administered to human subjects in malaria endemic areas. Therefore the safety of the vaccine in this population has yet to be established (in semi immune individuals). In addition, the discomforts of this study are those of having blood drawn from an arm vein, IM injection of the vaccine, and possible reactions to the vaccine. Drawing blood causes transient discomfort and may cause fainting. Infection at the site where blood will be drawn or where the vaccination is given is extremely unlikely. Bruising at the site of blood drawing may occur, but can be prevented or lessened by applying pressure for several minutes. Intramuscular injection may cause injection site pain, swelling, and redness. Immediate allergic reactions to vaccine, including anaphylaxis, are extremely rare (approximately 1 person in 4,000,000), and might occur as a skin rash, difficulty in breathing, fainting, drop in the blood pressure and death. Such reactions can usually be stopped by emergency medications administered by study personnel, if needed. Vaccine recipients may develop systemic reactions such as fever, headaches, body aches, and fatigue. These reactions are usually greatest within the first 24 to 72 hours after vaccination and last 1 to 2 days. Analgesics (e.g., aspirin or Tylenol®) and rest will generally relieve or moderate these symptoms. Other hypersensitivity reactions, including Arthus reactions, are also possible.

2.3.2 Known Potential Benefits

There are no direct benefits to subjects from participating in the study. However, volunteers may benefit altruistically by the knowledge that they are aiding in the malaria vaccine development effort. They may also receive medical attention beyond that which they normally receive, including the screening medical examination, periodic clinical laboratory examinations, and referral for treatment for medical problems that arise during the study. In addition, medical treatment will be provided for malaria illness for all volunteers during the study period, regardless of study-relatedness.

2.3.3 Provision of Study-Related Care

During this study, medical treatment, including hospitalization if necessary, will be provided to any volunteer who requires such treatment or hospitalization as a result of his or her participation in this study, as soon as such need is recognized. Any such acute care will be provided free of charge by the University Hospital. Volunteers will not receive any other compensation for any injury or problem, only medical care. Any disagreements or concerns about whether an event is considered related to the study and eligible for care will be resolved by the ISM. Any volunteer or prospective volunteer who is found to have diseases unrelated to the research and to malaria will be referred for health-care services as appropriate. DMID does not provide financial coverage for long-term care. This policy does not relinquish a volunteer's rights to seek compensation. Any volunteer who remains active in the study, but discontinues his/her vaccinations will continue to be seen for examinations and drawing of safety labs for the duration of the study.

Insurance: Insurance coverage for the trial will be provided from a local insurance company. The company undertakes to indemnify the institution (NMIMR) against any claims for negligent and /or non-negligent harm to any volunteer as a result of his/her participation in the study. Coverage will continue for 3 years from the time of enrolment.

3 OBJECTIVES

3.1 Study Objectives

The primary objective is to assess the safety and reactogenicity (tolerability) of ascending dosages of EBA-175 RII-NG vaccine among healthy semi immune adult subjects given in 3 doses at 0, 1 and 6 months by IM injection.
The secondary objective is to evaluate the immunogenicity of the EBA-175 RII-NG vaccine by measuring anti-EBA-175 RII-NG antibodies using enzyme-linked immunosorbent assay (ELISA), inhibition of *P. falciparum* growth *in vitro*, and inhibition of binding of EBA-175 RII-NG to red blood cells (RBCs)

3.2 Study Outcome Measures

3.2.1 Primary Outcome Measures

- The number of subjects experiencing severe (Grade 3) solicited injection site reactions within 14 days following vaccination.
- The number of subjects experiencing severe solicited systemic reactions (Grade 3) within 14 days following vaccination.
- The number of subjects experiencing severe (Grade 3) clinical laboratory values within 14 days following vaccination.
- The number of subjects spontaneously reporting adverse events considered associated with the vaccination that are severe (Grade 3) at any point during the study period.
- Serious adverse events considered associated with the vaccination reported at any point during the study period.

3.2.2 Secondary Outcome Measures

- Anti-EBA-175 RII-NG antibody level by ELISA at days 0, 14, 28, 42, 180 and 194..

- The number of subjects experiencing a 4-fold increase in Anti-EBA-175 RII-NG antibody level (ELISA) at days 14, 28, 42, 180 and 194 relative to baseline.
- Relative growth inhibition of *Pf* in human RBCs cultured *in vitro* in the presence of serum from immunized individuals at days 0, 14, 28, 42, 180 and 194.
- Relative binding inhibition of recombinant EBA-175 RII-NG to human RBCs *in vitro* in the presence of serum from immunized individuals at days 0, 14, 28, 42, 180 and 194.

4 STUDY DESIGN

The study is a single-center, placebo-controlled, randomized, dosage-escalation clinical trial to assess the safety of EBA-175 RII-NG vaccine administered in 3 doses intramuscularly in semi-immune adults. We propose to confirm the safety of the new vaccine by assessing the reactogenicity of the vaccine at 14 days after the second dose in the lower dosages before escalating to the next dosage level. Each dosage level group will include 18 subjects given vaccine and 2 subjects given placebo (normal saline) intramuscularly.

**Group**	**Vaccine Formulation**	**Number of Vaccine/Placebo**
A	5 µg EBA-175 + 500 µg aluminum phosphate adjuvant	18/2
B	20 µg EBA-175 + 500 µg aluminum phosphate adjuvant	18/2
C	80 µg EBA-175 + 500 µg aluminum phosphate adjuvant	18/2
Total		54/6

5 STUDY ENROLLMENT AND WITHDRAWAL

A total of 60 subjects between the ages of 18 and 40 years will be vaccinated (no allowance for replacements). Subjects will be recruited from the community at large in Legon and the surrounding suburbs of Accra. The University is home to a large student population (>20,000) while the suburbs of Haatso, East and West Legon, North Legon and Adenta have a substantial population base. These areas are served by the University Hospital which estimates the catchment population to be in the region of 100,000. Information regarding the study will be broadcast on the local FM station – Radio Universe – and also advertised in fliers at the hospital, on the campus and at strategic places in the suburbs. Interested persons will be instructed to contact the study team at the Clinical Trial Centre at the Noguchi Memorial Institute for Medical Research, on the campus of the university. All materials to be used in soliciting volunteers will be approved by the local Institutional Review Board (IRB) prior to their use.

This study will be performed as a randomized, double-blinded, dosage-escalation study. The first dosage cohort consists of 20 subjects: 18 will receive the malaria vaccine EBA-175 + 500 μg aluminum adjuvant while 2 will receive placebo control product. The subjects in the first cohort will be enrolled as follows: subject #1 followed by subject #2 with a minimum of a 30 minute waiting period between vaccinating subjects. This schedule will allow for assessment of immediate reactions of subject #1 prior to vaccinating subject #2. Following enrollment of subject #2, a minimum of a 2-day waiting period will be required prior to enrollment of the next six subjects. This schedule will allow for further reactogenicity assessment. The minimum 2-day waiting period will continue between each group of six subjects until the enrollment of 20 subjects into the first cohort has been met. This process will also be followed for Cohorts B and C.

The SMC will convene and make recommendations on dosage escalation and receipt of the third dose of the dosage under consideration, based on the safety data collected through day 14 after the completion of follow-up on the first 2 doses of a given dosage (6 weeks after dose 1).

Enrollment of additional subjects within any dosage cohort will not occur if a halting rule is met. Enrolled subjects who drop out of the study for any reason will not be replaced.

5.1 Subject Inclusion Criteria

1. Healthy males and healthy non-pregnant and non breastfeeding females between the ages of 18 and 40 years.
2. Females of childbearing potential must agree to practice adequate contraception through out the study and for 3 months after the third vaccination (including abstinence; hormonal contraception; condoms with spermicidal agents;) Males with female partners of childbearing age must agree to use condoms or other birth control.
3. Good health as determined by screening medical history, physical examination (PE), and routine laboratory assessments.
4. Willingness to comply with protocol requirements.
5. Ability to provide informed consent before any protocol procedures are performed.
6. Availability for follow-up for 12 months after the first immunization dose.

5.2 Subject Exclusion Criteria

1. Regular use of medications other than vitamins and contraceptives.
2. Current or recent (within the last 4 weeks prior to vaccination) treatment with parenteral, inhaled, or oral corticosteroids (intranasal steroids are acceptable), or other immunosuppressive agents, or chemotherapy.
3. History of splenectomy.
4. Abnormal screening laboratory values (see Appendix B). Any abnormal screening value for any screening test, will exclude the subject from the study. An exception to this rule is the glucose measurement. Random plasma glucose will be measured on all subjects during the screening visit. Values higher than 110mg/dl will be confirmed by a repeat fasting glucose measurement.
5. History of or current medical, occupational, social or family problems as a result of alcohol or illicit drug use by the volunteer.
6. History of moderate to severe mental illness, as defined by symptoms interfering with social or occupational function or suicidal thoughts/attempts.

7. History of receiving blood or blood products (such as blood transfusion, platelet transfusion, immunoglobulins, hyperimmune serum) in the previous 6 months.
8. Vaccination with a live vaccine within the past 30 days or with a non-replicating, inactivated, or subunit vaccine within the last 14 days.
9. Known hypersensitivity to components of the vaccine (EBA-175 RII-NG, sucrose, or aluminum adjuvant).
10. History of acute or chronic medical conditions including, but not limited to, disorders of the liver, kidney, lung, heart, or nervous system, or other metabolic and autoimmune /inflammatory conditions, sickle cell disease.
11. History of anaphylaxis or severe hypersensitivity reaction.
12. Severe asthma, as defined by an emergency room visit or hospitalization within the last 12 months.
13. Pregnant or breastfeeding women or women unwilling to use effective contraception during the study period.
14. Acute illness, including temperature >37.8 ^{o}C within one week prior to vaccination.
15. Positive serology for human immunodeficiency virus (HIV), hepatitis C virus (HCV), or hepatitis B surface antigen (HBsAg).
16. Concurrent participation in other investigational protocols or receipt of an investigational product within the previous 30 days.
17. Identification of any condition that, in the opinion of the investigator, would affect the ability of the subject to understand or comply with the study protocol or would jeopardize the safety or rights of a subject participating in the study.
18. History of malignancy, including hematologic and skin cancers, or known immunodeficiency syndrome.
19. **Pre-medication** with analgesic or antipyretic in the 6 hours prior to vaccination, or **planned** medication with analgesic or antipyretic in the 24 hours following vaccination. This criterion should not preclude subjects receiving such medication after vaccination if the need arises.

5.3 Treatment Assignment Procedures

5.3.1 Randomization Procedures

Randomization to either vaccine or placebo will be done online using the enrollment module of The EMMES Corporation AdvantageEDC^{SM} internet-based data entry system. The randomization codes will be included in the enrollment module for the trial. Each subject enrolled into the trial is assigned a treatment code after demographic and eligibility data have been entered into the system. The site will be provided with a treatment code list to be kept in a secure place with access permitted only to the vaccinator. The treatment assignment will be known only to the unblinded vaccinator. Internet access is very reliable at study center. A backup manual randomization procedure will be available in the event the internet or AdvantageEDC^{SM} are not available.

5.3.2 Masking Procedures

The vaccinator will have exclusive access to the treatment code list. After randomization of the subjects in AdvantageEDC^{sm}, the vaccinator will prepare the injection, and will mask the syringe with tape (vaccine or placebo, according to the key) in the vaccination room which is at the study site. No subjects or other personnel will be present at the time of preparation. When the injection is ready for administration, the subject will be called to the room and the injection given. The vaccinator will be the only unblinded individual involved in the trial. Personnel assessing reactogenicity of the vaccine will be blinded (within dosage group).

5.3.3 Reasons for Withdrawal

Subjects are free to withdraw from the study at any time. Subjects who have received vaccine or who developed an adverse event or serious adverse event will be encouraged to remain in the study to be followed for safety purposes. A study subject will be discontinued from receiving further investigational product if any clinical AE, laboratory abnormality, intercurrent illness, other medical condition or situation occurs that meets the exclusion criteria and is determined to be clinically significant by the PI in consultation with the DMID Medical Monitor, or if continued participation in the study would not be in the best interest of the subject. All exclusion and inclusion criteria will be reviewed before each vaccination. Any deviation from these criteria will reviewed to determine clinical significance by the PI in consultation with the DMID Medical Monitor and a decision will be made regarding additional vaccination.

All participants will be followed according to the prespecified schedule with principal, and perhaps secondary, outcome assessments, regardless of compliance, adverse effects, or other post-randomization observations—death and participant refusal excepted.

5.3.4 Handling of Subjects Who Discontinue Vaccination

If, for safety reasons, a subject is deemed by the investigators and/or safety monitoring committee (SMC) to be not eligible to receive the study product as per protocol, he/she will discontinue subsequent vaccinations and be followed for safety and immunogenicity through the next scheduled visits until a subsequent vaccination would have been scheduled and/or there has been resolution of any event which prompted discontinuation of the vaccinations. Subjects who discontinue the vaccinations or terminate their study participation early will not be replaced but every effort will be made to have them complete all study visits.

5.3.5 Termination of Study

The study termination form must be completed and signed by study personnel within 3 days of the subject completing the study or terminating his/her participation early.

6 STUDY INTERVENTION/INVESTIGATIONAL PRODUCT

6.1 Study Product Description

6.1.1 Acquisition

The study article will be shipped to the study site from DMID via the DMID Clinical Agent Repository at Fisher BioServices.

6.1.2 Formulation, Packaging, and Labeling

EBA-175 RII-NG malaria vaccine is supplied as a white, translucent, cloudy, non-particulate liquid suspension in single-dose clear glass vials pre-mixed with Adju-Phos aluminum phosphate adjuvant. Each 2-mL vial of EBA-175 RII-NG vaccine contains:

- 0.7 mL (0.5 mL per dose) EBA-175 RII-NG, at the required dose concentration
- 5% sucrose
- 1.0 mg/mL (0.5 mg/0.5 mL per dose) aluminum as aluminum phosphate adjuvant
- Sodium phosphate buffer (10 mM sodium phosphate and 150 mM sodium chloride)
- No preservative

The vials are labeled according to the concentration of EBA-175 RII-NG: 5 µg/0.5 mL Dose ; 20 µg/0.5 mL Dose ; 80 µg/0.5 mL Dose.

Normal saline placebo is supplied as single dose vials and is stored at room temperature.(between 20^0C and 25^0C).

6.1.3 Product Storage and Stability

Vials are to be stored refrigerated at a temperature of 2°C to 8°C. The placebo used will be normal saline (0.9% NaCl), stored in single dose vials at room temperature (between 20^0C and 25^0C). . Storage will be in a monitored refrigerator secured with an alarm.

6.2 Dosage, Preparation and Administration of Study Intervention/Investigational Product

Vaccination will be performed by a trained unblinded nurse. The study nurse who will vaccinate the subjects will not be involved in the assessment of vaccine reactogenicity. Each vaccine vial will be shaken gently to evenly mix the contents before use. A disposable 3.0 mL syringe with a sterile, disposable needle will be used to withdraw 0.5 mL from the vial for injection. Each dose is to be given by IM injection into the deltoid muscle. The subject will choose whether the injection will be administered into the right or left deltoid for the first dose. Subsequent injections may be administered in alternating arms. Placebo recipients will receive 0.5 mL of normal saline intramuscularly.

6.3 Modification of Study Intervention/Investigational Product for a Participant

If, for safety reasons or loss to follow-up, a subject is deemed by the investigators and/or SMC to be not eligible to receive the study product as per protocol, he/she will be terminated from subsequent vaccination and followed for safety and immunogenicity. No dose modification will be performed.

6.4 Accountability Procedures for the Study Intervention/Investigational Product(s)

The study article will be shipped via the DMID Clinical Agent Repository at Fisher BioServices to the study site. Vials of vaccine at each of the proposed 3 dosages will be sent to the study site. Unused vaccine vials will be stored unfrozen at a temperature of 2° to 8°C and discarded per institutional procedures following instructions from DMID after the study is completed. Used vials will also be stored until the study is completed and then discarded per institutional procedures.

6.5 Concomitant Medications/Treatments

Individuals on regular medications, with the exception of dietary supplements and contraceptives, are excluded from the study. However certain medications that are used

occasionally on an as-needed basis will be allowed in the study including, but not restricted to, analgesics, antihistamines and antipyretics.

7 STUDY SCHEDULE

7.1 Screening

The screening process will be performed by the investigators. Potential subjects will be provided with a verbal description of the study (purpose and study procedures). They will be asked if they have any questions and to read/sign the consent form. The consent form will be signed prior to the performance of any study procedures. The investigator will review the eligibility criteria and medical history with the subject. An oral temperature, height, weight, and blood pressure and pulse will be obtained. A targeted PE will be performed (lymph nodes, lung, heart, and abdomen). For females who are capable of bearing children, a urine pregnancy test will be done. A 10-mL blood sample will be collected from an arm vein to screen for health as follows: Hematology: complete blood count (CBC) with platelets, total white blood cell count (WBC) and differential, hemoglobin (Hgb); Chemistry: serum creatinine, electrolytes, alanine aminotransferase (ALT), aspartate aminotransferase (AST) and plasma glucose; Serology: HIV(which will be done by trained counselors at the Voluntary Counseling and Testing (VCT) center located at the Health Suppor Center of the Institute), HCV, HBsAg. A urine sample will be obtained for urinalysis. Medical history and concomitant medications will be recorded.

7.2 Enrollment/Baseline

See Section 8 and Appendix A.

7.3 Follow-Up

See Section 8 and Appendix A.

7.4 Final Study Visit

The final study visit is to be performed on Day 348.when serious adverse events (SAEs) will be assessed.

7.5 Early Termination Visit

If a subject withdraws from the study, refuses the receipt of the injection(s) but agrees to safety and immunogenicity follow-up, she/he will be followed for any occurrence of SAE during the observation period of their respective group through the next scheduled visits until a subsequent vaccination would have been scheduled. Blood for serology will be drawn as specified by the timetable of their respective group. If a subject withdraws from the study and refuses subsequent safety and immunogenicity follow-up, an attempt will be made to document AEs/SAEs at the early termination visit, and if the subject agrees, 35 mL of blood will be drawn for immunogenicity and safety assays.

7.6 Unscheduled Visit

Subjects may be asked to come in for additional clinic visits if the need arises (for example: follow-up on local or systemic AE or additional laboratory work-up). A supplemental visit source document will be filled and signed by the appropriate personnel. All unscheduled visits will be entered into the EMMES Corporation AdvantageEDC^{SM} internet data entry system on the appropriate eCRFs. Please see the Manual of Procedures for details.

8 STUDY PROCEDURES/EVALUATIONS

8.1 Clinical Evaluations

8.1.1 Screening Visit Day -28 to -1, Visit 1

- Potential subjects will be provided with a verbal description of the study (purpose and study procedures), and will be asked if they have any questions and to read/sign the consent form. The consent form will be signed prior to the performance of any study procedures.
- The investigator will discuss with the subject his/her medical history, study eligibility criteria and concomitant medication use.
- Vital signs (oral temperature, blood pressure, pulse) will be obtained.
- A targeted PE will be performed (lymph nodes, lungs, heart and abdomen) including height and weight.
- A 10-mL blood sample will be collected from an arm vein to screen for health as follows:
    - Hematology (4.5 mL): CBC with platelets, WBC count and differential, Hgb, hematocrit,.
    - Chemistry (5mL): serum creatinine, electrolytes, ALT, AST and plasma glucose
    - Serology (0.5mL): HIV, HCV, and HBsAg
- A urine sample will be obtained for urinalysis and pregnancy.

8.1.2 Day 0, Enrollment Visit 2

- Eligibility criteria will be reviewed with subjects. Vital signs (oral temperature, blood pressure, pulse) and interim medical history will be obtained.
- A targeted PE if indicated by the interim history.
- Concomitant medications will be recorded.
- Any spontaneous AEs that occur will be assessed.
- For females who are capable of bearing children, a urine pregnancy test will be done.
- A 35-mL blood sample for immunogenicity (25 mL) and safety laboratory (10 mL, CBC, and chemistry) testing will be collected from an arm vein prior to vaccination to provide a

baseline.

- A thick smear slide will be prepared from the 35ml blood sample and will be stored to be read for parasitemia at a later time if necessary. Refer to section 8.2.1
- A urine specimen will be collected for urinalysis.
- Enrolled subjects will be assigned to receive placebo or EBA-175 RII-NG vaccine using the dosage specified by their group (A→C, sequentially).
- Subjects will be observed in the clinic for at least 30 minutes following vaccination. The vaccination site will be examined, and the subject will be questioned on the presence of any localized or generalized reactogenicity symptoms. Vital signs (oral temperature, blood pressure, pulse) will be repeated.
- Subjects will be provided with a memory aid, a clear plastic ruler, and a digital thermometer, and will be instructed in their use prior to discharge from the clinic. Subjects will monitor temperature once a day and if subject feels feverish.
- Subjects will be instructed to notify the study center if they develop an oral temperature of 37.8^{o}C or higher or if they develop any severe reactions following vaccination.

8.1.3 Day 2 (± 1), Visit 3

- Subjects will be evaluated in the clinic 2 days after immunization. An oral temperature will be taken, and information regarding systemic and local reactogenicity will be solicited and reviewed. An examination of the vaccination site will be performed.
- A targeted PE will be performed if indicated by the subject's interim medical history.
- Concomitant medications will be reviewed and updated if applicable.
- Any spontaneous AEs that have occurred since the last visit will be assessed.
- Subjects' memory aids will be reviewed for accuracy and completeness.
- Subjects will be reminded to record their oral temperature, vaccination site symptoms and signs, and systemic symptoms for the 14-day period after vaccination.
- Subjects will be reminded to notify the study center if they develop an oral temperature of 37.8^{o}C or higher during the week after vaccination, or if they develop any severe reactions during the study.

8.1.4 Day 7 (± 2), Visit 4

- Subjects will be evaluated in the clinic 7 days after immunization. An oral temperature will be taken, and information regarding systemic and local reactogenicity will be solicited and reviewed. An examination of the vaccination site will be performed.
- A targeted PE will be performed, if indicated by the subject's interim medical history.
- Concomitant medications will be reviewed and updated, if applicable.
- Any spontaneous AEs that have occurred since the last visit will be assessed.
- Subjects' memory aids will be reviewed for accuracy and completeness.
- Subjects will be reminded to record their oral temperature, vaccination site symptoms and signs, and systemic symptoms for the 14-day period after vaccination.
- Subjects will be reminded to notify the study center if they develop an oral temperature of 37.8^{o}C or higher during the week after vaccination, or if they develop any severe reactions during the study.

8.1.5 Day 14 (± 3), Visit 5

- Subjects will be evaluated in the clinic 14 days after immunization. An oral temperature will be taken, and information regarding systemic and local reactogenicity will be solicited and reviewed. An examination of the vaccination site will be performed.
- A targeted PE will be performed if indicated by the subject's interim medical history.
- Concomitant medications will be reviewed and updated, if applicable.
- Any spontaneous AEs that have occurred since the last visit will be assessed.
- Subjects' memory aids will be reviewed for accuracy and completeness and collected.
- A 35-mL blood sample will be collected for immunogenicity and safety laboratory (CBC, and chemistry) testing. A urine specimen will also be collected for testing.

8.1.6 Day 28 (± 3 days), Visit 6

- Eligibility criteria will be reviewed with subjects. An oral temperature, blood pressure, pulse and interim medical history will be obtained.
- A targeted PE if indicated by the interim history.

- Concomitant medications will be recorded.
- Any spontaneous AEs that have occurred since the last visit will be assessed.
- Information regarding SAEs will be solicited and recorded.
- For females, a urine pregnancy test will be performed.
- A 35-mL blood sample for immunogenicity (25 mL) and safety laboratory testing (10 mL) will be collected from an arm vein prior to vaccination. A urine specimen will also be collected for testing.
- A thick smear slide will be prepared from the 35mL blood sample and will be stored to be read for parasitemia at a later time if necessary. Refer to section 8.2.1
- The second dose of placebo or EBA-175 RII-NG vaccine will be given at the same dosage used on Day 0.
- Subjects will be observed in clinic for at least 30 minutes following vaccination. The vaccination site will be examined, and the subject will be questioned on the presence of any localized or generalized reactogenicity symptoms. Vital signs (oral temperature, blood pressure, pulse) will be repeated.
- Subjects will be provided with a memory aid, a clear plastic ruler, and a digital thermometer, and will be instructed in their use prior to discharge from the clinic.
- Subjects will be instructed to notify the study center if they develop an oral temperature of 37.8^{o}C or higher or if they develop any severe reactions following vaccination.

8.1.7 Day 30 (± 1), Visit 7

- Subjects will be evaluated in the clinic 2 days after their second immunization. An oral temperature will be taken, and information regarding systemic and local reactogenicity will be solicited and reviewed. An examination of the vaccination site will be performed.
- A targeted PE will be performed if indicated by the subject's interim medical history.
- Concomitant medications will be reviewed and updated, if applicable.
- Any spontaneous AEs that have occurred since the last visit will be assessed.
- Subjects' memory aids will be reviewed for accuracy and completeness.
- Subjects will be reminded to record their oral temperature, vaccination site symptoms and signs, and systemic symptoms for the 14-day period after vaccination.

- Subjects will be reminded to notify the study center if they develop an oral temperature of 37.8^{o}C or higher during the week after vaccination, or if they develop any severe reactions during the study.

8.1.8 Day 35 (± 2), Visit 8

- Subjects will be evaluated in the clinic 7 days after their second immunization. An oral temperature will be taken, and information regarding systemic and local reactogenicity will be solicited and reviewed. An examination of the vaccination site will be performed.
- A targeted PE will be performed if indicated by the subject's interim medical history.
- Concomitant medications will be reviewed and updated, if applicable.
- Any spontaneous AEs that have occurred since the last visit will be assessed.
- Subjects' memory aids will be reviewed for accuracy and completeness.
- Subjects will be reminded to record their oral temperature, vaccination site symptoms and signs, and systemic symptoms for the 14-day period after vaccination.
- Subjects will be reminded to notify the study center if they develop an oral temperature of 37.8^{o}C or higher during the week after vaccination, or if they develop any severe reactions during the study.

8.1.9 Day 42 (± 3), Visit 9

- Subjects will be evaluated in the clinic 14 days after their second immunization. An oral temperature will be taken, and information regarding systemic and local reactogenicity will be solicited and reviewed. An examination of the vaccination site will be performed.
- A targeted PE will be performed if indicated by the subject's interim medical history.
- Concomitant medications will be reviewed and updated, if applicable.
- Any spontaneous AEs that have occurred since the last visit will be assessed.
- Subjects' memory aids will be reviewed for accuracy and completeness and collected.
- A 35-mL blood sample will be collected for immunogenicity and safety laboratory testing. A urine specimen will also be collected for testing.

8.1.10 Day 180 (± 14), Visit 10

- Eligibility criteria will be reviewed with subjects. An oral temperature, blood pressure, pulse and interim medical history will be obtained.
- A targeted PE if indicated by the interim medical history.
- Concomitant medications will be recorded.
- Any spontaneous AEs that have occurred since the last visit will be assessed.
- For females, a urine pregnancy test will be performed.
- A 35-mL blood sample for immunogenicity (25 mL) and safety laboratory testing (10 mL) will be collected from an arm vein prior to vaccination. A urine specimen will also be collected for testing.
- A thick smear slide will be prepared from the 40mL blood sample and will be stored to be read for parasitemia at a later time if necessary. Refer to section 8.2.1
- The third dose of placebo or EBA-175 RII-NG vaccine will be given at the same dosage used on Days 0 and 28.
- Subjects will be observed in clinic for at least 30 minutes following vaccination. The vaccination site will be examined, and the subject will be questioned on the presence of any localized or generalized reactogenicity symptoms. Vital signs (oral temperature, blood pressure, pulse) will be repeated.
- Subjects will be provided with a memory aid, a clear plastic ruler, and a digital thermometer, and they will be instructed in their use prior to discharge from the clinic.
- Subjects will be instructed to notify the study center if they develop an oral temperature of 37.8 ^{0} C or higher or if they develop any severe reactions following vaccination.

8.1.11 Day 182 (± 1), Visit 11

- Subjects will be evaluated in the clinic 2 days after their third immunization. An oral temperature will be taken, and information regarding systemic and local reactogenicity will be solicited and reviewed. An examination of the vaccination site will be performed.
- A targeted PE will be performed if indicated by the subject's interim medical history.
- Concomitant medications will be reviewed and updated, if applicable.
- Any spontaneous AEs that have occurred since the last visit will be assessed.

- Subjects' memory aids will be reviewed for accuracy and completeness.
- Subjects will be reminded to record their oral temperature, vaccination site symptoms and signs, and systemic symptoms for the 14-day period after vaccination.
- Subjects will be reminded to notify the study center if they develop an oral temperature of 37.8°C or higher during the week after vaccination, or if they develop any severe reactions during the study.

8.1.12 Day 187 (± 2), Visit 12

- Subjects will be evaluated in the clinic 7 days after their third immunization. An oral temperature will be taken, and information regarding systemic and local reactogenicity will be solicited and reviewed.. An examination of the vaccination site will be performed.
- A targeted PE will be performed if indicated by the subject's interim medical history.
- Concomitant medications will be reviewed and updated, if applicable.
- Any spontaneous AEs that have occurred since the last visit will be assessed.
- Subjects' memory aids will be reviewed for accuracy and completeness.
- Subjects will be reminded to record their oral temperature, vaccination site symptoms and signs, and systemic symptoms for the 14-day period after vaccination.
- Subjects will be reminded to notify the study center if they develop an oral temperature of 37.8°C or higher during the week after vaccination, or if they develop any severe reactions during the study.

8.1.13 Day 194 (± 3), Visit 13

- Subjects will be evaluated in the clinic 14 days after their third immunization. An oral temperature will be taken, and information regarding systemic and local reactogenicity will be solicited and reviewed. An examination of the vaccination site will be performed.
- A targeted PE will be performed if indicated by the subject's interim medical history.
- Concomitant medications will be reviewed and updated, if applicable.
- Any spontaneous AEs that have occurred since the last visit will be assessed.
- Subjects' memory aids will be reviewed for accuracy and completeness and collected.
- A 35-mL blood sample will be collected for immunogenicity and safety laboratory testing. A

urine specimen will also be collected for urinalysis.

8.1.14 Day 208 (± 7) Visit 14

- Subjects will be evaluated in the clinic 28 days after their third immunization. An oral temperature will be taken, and any spontaneous AEs that have occurred since the last visit will be solicited and reviewed. An examination of the vaccination site will be performed
- A targeted PE will be performed if indicated, by the subject's interim medical history.
- Concomitant medications will be reviewed and updated, if applicable.
- Any spontaneous AEs that have occurred since the last visit will be assessed.
- A 10-mL blood sample will be collected for safety laboratory testing. A urine specimen will also be collected for urinalysis (and pregnancy testing for female subjects).

8.1.15 (Day 258 ± 21) Visit 15

Subjects will be evaluated in the clinic approximately 3 months (± 21 days) after their last immunization. An oral temperature, BP will be taken and any spontaneous AEs that have occurred since the last visit will be assessed.

8.1.16 Day 348 (± 21) Visit 16

Subjects will be evaluated in the clinic approximately 6 months (± 21 days) after their last immunization. An oral temperature and BP will be taken and any spontaneous AEs that have occurred since the last visit will be assessed.

8.2 Laboratory Evaluations

8.2.1 Clinical Laboratory Evaluations

- Hematology: hemoglobin, hematocrit, WBC with differential, platelet count (4.5 mL EDTA anticogulated blood).
- Chemistry: Serum ALT, AST, electrolytes, creatinine and plasma glucose (5 mL of blood in serum separator tube (SST).
- Serology: HIV, HBsAg, HCV antibodies (0.5 mL of blood in SST).

- A thick smear slide will be prepared prior to each vaccination and will be stored to be read for parasitemia at a later time in the event that a safety concern arises regarding a possible association between vaccination and concomitant asymptomatic parasitemia.
- Urinalysis: dipstick for blood, WBC and proteins (10 mL random void: clean catch urine in a plastic cup; if dipstick is abnormal, urine microscopy will be performed) and pregnancy.
- All clinical laboratory assays are performed at the Clinical Safety Laboratory, NMIMR.
- HIV testing will be performed at the VCT center of NMIMR or the University of Ghana Hospital.

8.2.2 Special Assays or Procedures

To assess the immunogenicity of the vaccine, 25 mL of blood will be drawn from subjects into serum separator tubes on the following study days: 0, 14, 28, 42, 180 and 194 for analysis. The assays to be performed include ELISA to assess anti-EBA-175 RII-NG antibody production and growth inhibition of *Pf* by serum in an *in vitro* assay. Two laboratories will be responsible for the immunogenicity assessment. NMIMR will be the primary laboratory to assess anti-EBA-175 RII-NG antibody production by ELISA while the Laboratory of Malaria and Vector Research National Institute of Allergy and Infectious Diseases, National Institutes of Health will be responsible for the growth inhibition assay. An ongoing blood draw protocol on assay validation (*Quality Control of Immunological Reagents and Validation of Modifications and Improvements to Immunological Assays in Support of Malaria Vaccine Trials*) will be used to standardize for EBA-175 RII-NG ELISA between the two laboratories.

8.2.2.1 Instructions for Serum Specimen Preparation, Handling and Storage

Following collection, blood will be allowed to sit a minimum of 30 minutes. Then it will be centrifuged in the serum separator tube according to manufacturer’s specifications. Under sterile conditions, the serum will be collected and aliquoted according to study procedures. Labeled aliquots will be placed in the appropriate fiberboard cryoboxes and stored in local inventory. The filled cryoboxes will be stored in a -70°C to -90°C freezer that is monitored by a central alarm system. Half of the aliquoted plasma samples will be shipped on dry ice to Laboratory of Malaria and Vector Research National Institute of Allergy and Infectious Diseases National Institutes of Health, USA using approved shipping procedures. Specimens will be labeled with bar codes for tracking and shipments will be managed by the EMMES Corporation using GlobalTrace^{SM} Specimen Tracking System software.

9 ASSESSMENT OF SAFETY

9.1 Specification of Safety Parameters

Vaccine reactogenicity will be assessed via the memory aid and in-clinic assessments through 28 days after the last dose.

9.2 Methods and Timing for Assessing, Recording, and Analyzing Safety Parameters

9.2.1 Adverse Events

Adverse Event: International Conference on Harmonisation (ICH) E6 Good Clinical Practice Guidelines (GCP) defines an adverse event (AE) as any untoward medical occurrence in a patient or clinical investigation subject administered a pharmaceutical product regardless of its causal relationship to the study treatment. An AE can therefore be any unfavorable and unintended sign (including an abnormal laboratory finding), symptom, or disease temporally associated with the use of medicinal (investigational) products. The occurrence of an AE may come to the attention of study personnel during study visits and interviews or by a study recipient presenting for medical care. All AEs must be graded for intensity and relationship to study product. Solicited local and systemic adverse events, reactogenicities, collected through the memory aid during 14 days following vaccination will be considered associated with vaccination.

Severity of Event: All AEs will be assessed by the investigator using a protocol-defined grading system (Appendices B and C). For events not included in the protocol defined grading system, the following guidelines will be used to quantify intensity.

- Mild: events require minimal or no treatment and do not interfere with the subject’s daily activities.
- Moderate: events result in a low level of inconvenience or concern with the therapeutic measures. Moderate events may cause some interference with functioning.
- Severe: events interrupt a subject’s usual daily activity and may require systemic drug therapy or other treatment. Severe events are usually incapacitating.

- Life-threatening: Any adverse drug experience that places the patient or subject, in the view of the investigator, at immediate risk of death from the reaction as it occurred, ie, it does not include a reaction that had it occurred in a more severe form, might have caused death.

Adverse events characterized as intermittent require documentation of onset and duration of each episode.

Relationship to Study Products: The investigator's assessment of an AE's relationship to study drug is part of the documentation process, but it is not a factor in determining what is or is not reported in the study. If there is any doubt as to whether a clinical observation is an AE, the event should be reported. All AEs must have their relationship to the study product assessed using the following terms: associated or not associated. In a clinical trial, the study product must always be suspect. To help assess, the following guidelines are used:

- Associated - The event is temporally related to the administration of the study product and no other etiology explains the event.
- Not Associated - The event is temporally independent of study product and/or the event appears to be explained by another etiology.

9.2.2 Reactogenicity

For this study, reactogenicity information will be collected in the memory aid for 14 days and in the clinic from the time the subject receives the injection until 28 days after immunization. Events continuing beyond 14 days post-vaccination will be followed as an AE until the time of resolution or stabilization. Solicited reactogenicity information will include:

- Laboratory safety parameters as shown in Appendix B.
- Fever: An oral temperature of 37.8°C (100°F) or above will be considered fever. Fever severity will be scored as follows:

  0 = Oral temperature <100°F (<37.8°C);

  1 = Mild (100°F-101°F, or 37.8°C-38.3°C);

  2 = Moderate (101.1°F-103°F, or 38.4°C-39.4°C);

  3 = Severe (>103°F, or >39.4°C).

- Vaccination site symptoms/signs (pain, tenderness, erythema, edema, and induration): Reactogenicity at the site of injection (erythema, induration, edema) will be measured in

millimeters. Subjects will be provided with instructions and a diary to record any vaccine site symptoms in detail (Appendix C).

- Systemic symptoms: Systemic symptoms include fever, chills, arthralgias, fatigue, malaise, myalgia, headache, nausea and vomiting. Reactogenicity will be evaluated using memory aids to solicit information about specific local and systemic symptoms. Reactogenicity will be analyzed using the following grading systems:

  0 = Absence of the indicated symptom;
  1 = Mild (awareness of a symptom but the symptom is easily tolerated);
  2 = Moderate (discomfort enough to cause interference with usual activity); and
  3 = Severe (incapacitating; unable to perform usual activities; requires absenteeism or bed rest);
  4 = Life-threatening.

Otherwise, all unsolicited AEs will be recorded on the AE page of the case report forms (CRFs).

9.2.3 Serious Adverse Event

A Serious Adverse Event (SAE) is defined as an AE meeting one of the following conditions:

- Death during the period of protocol defined surveillance
- Life-threatening event (defined as a subject at immediate risk of death at the time of the event)
- An event requiring inpatient hospitalization or prolongation of existing hospitalization during the period of protocol defined surveillance
- Results in congenital anomaly or birth defect
- Results in a persistent or significant disability/incapacity

Serious adverse event information will be collected from Day 0 through Day 348 (± 21 days).

Any other important medical event that may not result in death, be life-threatening, or require hospitalization, may be considered a serious adverse experience when, based upon appropriate medical judgment, the event may jeopardize the subject and may require medical or surgical intervention to prevent one of the outcomes listed above. Examples of such medical events include allergic bronchospasm requiring intensive treatment in an emergency room or at home, blood dyscrasias or convulsions that do not result in inpatient hospitalization, or the development of drug dependency or drug abuse.

9.2.4 Procedures to be Followed in the Event of Abnormal Laboratory Test Values or Abnormal Clinical Findings

Abnormal laboratory values and clinical findings that occur during the study observation period and that are deemed to be associated with the study will be followed until resolution. SAE's will be followed until resolution or until the condition is considered chronic.

9.3 Reporting Procedures

Adverse Events: Adverse events including local and systemic reactions not meeting the criteria for "serious adverse events" will be captured on the appropriate CRF. Information to be collected for spontaneously reported AEs includes event description, time of onset, investigator assessment of severity, relationship to study product, time of resolution of the event, seriousness, and outcome. All AEs occurring while on study must be documented appropriately regardless of relationship. All AEs will be followed to adequate resolution. An event is reportable as associated with the vaccine if the possibility of a relationship of the vaccine to the event cannot be ruled out. Any medical condition that is present at the time that the subject is screened should be considered as baseline and not reported as an AE. However, if it deteriorates at any time during the study, it should be recorded as an AE.

9.3.1 Serious Adverse Event Detection and Reporting

All SAEs that occur during the study will be

- recorded on the appropriate SAE case report form;
- followed through resolution by a study physician;
- reviewed by a study physician.
- Assessed by an Independent Safety Monitor whose assessment will be shared with DMID

Any AE considered serious by the principal investigator (PI) or subinvestigator or that meets the aforementioned criteria must be submitted on an SAE form to the DMID Pharmacovigilance Group, at the following address:

DMID Pharmacovigilance Group
Clinical Research Operations and Management Support (CROMS)
6500 Rock Spring Dr. Suite 650
Bethesda, MD 20814, USA
SAE Hot Line: 1-800-537-9979 (US) or 1-301-897-1709 (outside US)
SAE FAX Phone Number: 1-800-275-7619 (US) or 1-301-897-1710 (outside US)
SAE Email Address: PVG@dmidcroms.com

The study clinician will complete a SAE form within the following timelines:

- All deaths and life-threatening events, whether associated or not associated, will be recorded on the SAE form and sent by fax within 24 hours of site awareness of the event.
- Serious adverse events other than death or life-threatening events, regardless of relationship, will be reported via fax by the site within 24 hours of becoming aware of the event.

Other supporting documentation of the event may be requested by the pharmacovigilance contractor and should be provided as soon as possible.

All SAEs will be followed until satisfactory resolution or until the PI or subinvestigator deems the event to be chronic or the subject to be stable.

All SAEs will be reported as required to the site IRB. SAE data will be entered into the EMMES Corporation AdvantageEDC^{SM} at NMIMR within one business day of data acquisition.

9.3.2 Regulatory Reporting for Studies Conducted Under DMID-Sponsored IND

Following notification from the investigator, DMID, the IND sponsor, will report events that are both serious and unexpected and that are associated with study product(s) to the Food and Drug Administration within the required timelines as specified in 21 CFR Part 312.32: fatal and life-threatening events within 7 calendar days (by phone or fax) and all other SAEs in writing within 15 calendar days. All serious events designated as “not associated” to study product(s), will be reported to the Food and Drug Administration at least annually in a summary format.

9.3.3 Reporting of Pregnancy

In the event that a subject becomes pregnant, the pregnancy will be reported to the independent safety monitor (ISM), sponsor and NMIMR IRB within 24 hours after the site becoming aware of the occurrence. The subject will be discontinued from receiving subsequent injections and will be in safety follow-up only. The outcome of the pregnancy will be documented and reported to the monitor, the sponsor, and the NMIMR IRB. A Pregnancy Reporting Form, provided by EMMES will be used and entered into EMMES data system. Any pregnancy that occurred within 3 months after the last immunization will be followed and the infant will be assessed for 6 months after delivery.

9.4 Type and Duration of Follow-Up of Subjects after Adverse Events

All AEs will be followed until resolution. All SAEs will be followed until resolution or until the condition is considered chronic.

9.5 Halting Rules

Subject safety data will be reviewed on an ongoing basis. Memory aid and safety laboratory data will be entered into the EMMES Corporation AdvantageEDC^{SM} at NMIMR within 2 business days of data acquisition (e.g., receiving the memory aid information or the laboratory reports). If any of the following events occur, then enrollment will be stopped and data will be reviewed. The EMMES Corporation together with NMIMR data management personnel will monitor the occurrence of the events listed below and notify the investigators and NIH/NIAID/DMID if any of the stopping criteria are met. If internet service is not available, EMMES will be called and/or FAX'd. A decision to proceed or to terminate enrollment, vaccinations, or the trial will be made in consultation with the SMC (including the independent safety monitor), the NIH/NIAID/DMID, and the clinical investigators.

1. If 2 subjects in any single vaccine group experience severe (Grade 3) vaccination site pain within 1 week following vaccination;
2. If 2 subjects in any single vaccine group experience moderate (Grade 2) or higher fever that is associated with the vaccination within 1 week following vaccination;

3. If 2 subjects experience a spontaneously reported adverse event that is graded to be severe (Grade 3) or higher and is associated with vaccination at any time during the follow-up period;
4. If 2 subjects experience a systemic solicited reactogenicity that is graded severe (Grade 3) or higher (other than vaccine-related fever as defined above) within 14 days after vaccination
5. If 1 subject experiences an SAE judged by an investigator to be associated with vaccination at any point during follow-up;
6. If 1 subject develops a laboratory value that is considered severe (grade 3) or life-threatening and that is judged to be associated with vaccination at any point during follow-up;
7. Any other observation occurs that in the opinion of the PI or NIH/NIAID results in a recommendation to halt enrollment and or further vaccinations.

During clinic visits, the study personnel will ascertain that the reported severity of an AE by the subject meets the predefined criteria of the study of grading the severity of AEs.

9.6 Safety Oversight

An SMC will be assembled to review the safety data as they are collected. The SMC meets the specifications set forth in the DMID standard operating procedures and will have an established Charter.

The SMC will convene and make recommendations on dosage escalation and receipt of the third dose of the dosage under consideration, based on the safety data collected through day14 after the completion of follow-up on the first 2 doses of a given dosage (6 weeks after dose 1). The SMC receives the blinded safety data summaries on the first 2 doses of each cohort and will be reviewed in open session. If necessary, unblinded data will be available for review by the SMC in a closed session. The safety data will be compiled by the EMMES Corporation. Based on the recommendations of the SMC, the decision will be made regarding dosage escalation.

.,

***Ad Hoc* Meetings of SMC:** The SMC may convene an *ad hoc* meeting to discuss any issue of safety raised by an investigator, the sponsor, or a member of the SMC or if a halting rule is met. At the discretion of the investigators, the sponsors and SMC members a non-serious AE that is:

1) Associated with the product

2) Does not meet the stopping rules criteria

may be considered as a trigger for an *ad hoc* SMC meeting to assess the safety of the product, without resulting in halting the enrollment or further vaccinations of the trial.

An Independent Safety Monitor (ISM) will be assigned for the study site. The ISM is a physician with relevant expertise whose primary responsibility is to provide independent safety monitoring in a timely fashion. The ISM will review SAEs and other adverse events as needed and provide an independent assessment to DMID. The ISM will be a member of the SMC.

10 CLINICAL MONITORING STRUCTURE

10.1 Site Monitoring Plan

Site monitoring will be conducted to ensure that human subject protection, study procedures, laboratory procedures, study intervention administration, and data collection processes are of high quality and meet sponsor, Good Clinical Practice (GCP)/ICH, and regulatory guidelines, and that the study is conducted in accordance with the protocol and sponsor standard operating procedures. DMID, the sponsoring agency, or its designee will conduct site monitoring visits as detailed in the monitoring plan or in the manual of procedures. Site monitoring visits will be made at standard intervals as defined by DMID and may be made more frequently as directed by DMID. Site monitoring visits will include, but are not limited to, review of regulatory files, accountability records, case report forms, informed consent forms, medical and laboratory reports, and protocol compliance. Clinical Site Monitors will meet with investigators to discuss any problems and actions to be taken and to document visit findings and discussions.

11 STATISTICAL CONSIDERATIONS

11.1 Study Overview and Design

The study population will consist of semi-immune healthy young adults, between the ages of 18 and 40 years. The study is a single-center, placebo-controlled, randomized, dosage-escalating study to assess the safety of EBA-175 RII-NG vaccine administered in 3 doses intramuscularly. Clinic staff and study participants will be masked to treatment assignment (vaccine or placebo).

The primary objective of the study is to assess the safety and reactogenicity (tolerability) of ascending dosages of EBA-175 RII-NG among healthy subjects given 3 IM doses at 0, 1 and 6 months. The secondary objective of the study is to analyze the immunogenicity of the vaccine by measuring anti-EBA-175 RII-NG antibodies using ELISA, inhibition of binding of EBA-175 RII-NG to RBCs and inhibition of *Pf* growth *in vitro*.

The Primary outcomes and accompanying confidence intervals will be tabulated and presented graphically by dose group. In addition, the following more detailed results will be presented:

1. Summarizing the frequency of specific local and systemic reactions by days following vaccination.
2. Line listing of individual clinical and laboratory AEs as classified by immediate (within the first 30 minutes), systemic, and local will be displayed in tabular format.
3. AEs will be summarized by MedDRA System Organ Class, MedDRA Preferred Term, severity and relationship to vaccine .

Secondary outcomes will be presented graphically and in tables by dose group. Geometric Mean Titer of Anti-EBA-175 RII-NG antibody level by ELISA and accompanying 95% confidence intervals will be calculated.

We propose to confirm the safety of the study vaccine by assessing the reactogenicity of the vaccine at 14 days after the second dose in the lower dosages before escalating to the next dosage level. Each dosage level group will include 18 subjects given vaccine and 2 subjects given placebo (normal saline) intramuscularly.

11.2 Sample Size Considerations

The sample size for each dosage and route group is 18. (An additional 2 subjects will receive placebo for each dosage and route group.) This small number is selected to obtain preliminary

safety information on a small cohort of subjects before proceeding to larger trials. If no subject(s) within a dose/route group experiences an AE that meets a halting criterion, and study accrual is completed, the upper bound for a 1-sided 95% confidence interval for the probability of such an event is 0.15. Missing data will not be replaced. The proposed sample size will provide pilot data on secondary outcomes and will not assure adequate power to reject secondary hypothesis of dose-related immunogenic response unless that response is very large.

11.3 Final Analysis Plan

This study, like other Phase 1 studies, is exploratory rather than confirmatory; its purpose is to estimate event rates and patterns of immune responses rather than to test formal statistical hypotheses. Estimates will be presented with their 95% confidence intervals. Descriptive approaches will be used to meet the protocol objectives as stated in this protocol. Results will be presented in tabular format, as well as graphically when appropriate.

Formal comparisons between vaccine and placebo groups will not be made. The control group is primarily a bias and laboratory control. For the purposes of collecting pilot data and planning of potential future trials, some comparisons of secondary outcomes may be made with the placebo groups although the study is not powered to detect small to moderate differences.

The primary analysis will be conducted on data collected until Day 208 (28 days following the third immunization) for each dosage cohort. The data generated at this time will be used for decision making related to the product clinical development plan. The study will continue in a single-blind manner for additional safety surveillance. This additional information will be appended to the study report.

12 SOURCE DOCUMENTS AND ACCESS TO SOURCE DATA/DOCUMENTS

Electronic CRFs (eCRFs) will be supplied by the EMMES Corporation under contract to the NIAID, and a remote data entry system will be used. Data collection forms derived from the eCRFs will be made available on the project website for Source Document Workbooks (SDWs). The SDW for each subject will be maintained at the site. All SDWs will be filled out completely by appropriate study personnel.

The site will permit authorized representatives of the DMID, its designees, and appropriate regulatory agencies to examine (and when required by applicable law, to copy) clinical records for the purposes of quality assurance reviews, audits and evaluation of the study safety and progress. These representatives will be permitted access to all source data.

13 QUALITY CONTROL AND QUALITY ASSURANCE

NMIMR established a QA Unit in 2003, which reports to the Director of NMIMR. The Unit establishes policy and oversees practices to ensure that systems are in place for all work requirements, and that conformance standards are implemented to reduce variance. The operating premise for the Unit is that quality should be built into every process from the beginning, and that all tasks should be performed to meet or exceed regulatory requirements. Responsibilities include: assuring the availability of resources necessary to support GCPs; inspecting and approving Standard Operating Procedures (SOPs) and Study Specific Procedures (SSPs); examination of internal QA audit findings; determination of any action necessary to correct deficiencies; and, oversight of clinical laboratories used to assess safety of vaccines administered to volunteers. The Clinical Trials Unit of NMIMR will prepare a quality control and quality assurance plan based on this protocol. The plan will be made available at DMID and on site, upon request.

DMID-designated clinical monitors will verify that the clinical trial is conducted and data are generated, documented (recorded), and reported in compliance with the protocol, Good Clinical Practice, and the applicable regulatory requirements. Clinical monitoring reports will be submitted to DMID

The investigational site will provide direct access to all trial-related sites, source data/documents, and reports for the purpose of monitoring and auditing by the sponsor, and inspection by local and regulatory authorities.

The Statistical and Data Coordinating Center (SDCC) at The EMMES Corporation will implement quality control procedures beginning with the AdvantageEDC^{SM} data entry system and generate data quality control checks that will be run on the database. Any missing data or data anomalies will be communicated to the investigators for prompt clarification and resolution.

14 ETHICS/PROTECTION OF HUMAN SUBJECTS

14.1 Ethical Standard

The investigator will ensure that this study is conducted in full conformity with the principles set forth in the Helsinki Declaration of Helsinki, “The Belmont Report: Ethical Principles and Guidelines for the Protection of Human Subjects of Research of the US National Commission for the Protection of Human Subjects of Biomedical and Behavioral Research (April 18, 1979)” and codified in 45 CFR Part 46 and/or the ICH E6; 62 Federal Regulations 25691 (1997).

14.2 Institutional Review Board

The protocol, informed consent, protocol amendments, investigator brochures and advertisement materials will be submitted to NMIMR IRB for review and approval. The trial start will be contingent upon local IRB approval. All subsequent protocol amendments and SAEs will be submitted to the IRB in a timely manner.

14.3 Informed Consent Process

The informed consent process is initiated prior to the individual’s agreement to participate in the study and continuing throughout the individual’s study participation. Extensive discussion of risks and possible benefits of this therapy will be provided to the subjects. Consent forms describing in detail the study interventions, study procedures and risks are given to the subject and written documentation of informed consent is required prior to starting the screening process. Consent forms will be IRB approved and the subject will be asked to read and review the document. Upon reviewing the document, the investigator will explain the research study to the subject and answer any questions that may arise. The subjects will sign the informed consent document prior to any procedures being performed specifically for the study. The subjects may withdraw consent at any time throughout the course of the trial. The rights and welfare of the subjects will be protected by emphasizing to them that the quality of their medical care will not be adversely affected if they decline to participate in this study. A signed and dated copy of the consent form will be given to each subject for his/her records. Each subject will be asked to state his/her date of birth to verify age prior to completing the informed consent process.

14.4 Exclusion of Women, Minorities, and Children (Special Populations)

The study population consists of healthy young adults, including women. However, children, prisoners, and disabled individuals are not included in the study because the risk/benefit ratio is currently unknown.

14.5 Volunteer Recompense

All volunteers will receive the GHC (Ghana Cedi) equivalent of $25.00 ,USD for every study visit for study-related costs such as transportation and childcare, and inconvenience and time spent, and GHC (Ghana Cedi) equivalent of $15.00 USD for returning your completed diary. The total amount received depends on study completion; Full participation is the GHC (Ghana Cedi) equivalent of $415.00 USD. This modest amount is not so large as to induce prospective volunteers to consent to this study against their better judgment, but will help reimburse study related costs and time missed away from work and family due to study visits. In the case that a volunteer withdraws him/herself from the study for any reason or is withdrawn by the Principal Investigator for any reason, including health-related reasons, he or she will be paid in proportion to the amount of study participation.

Insurance: Insurance coverage for the trial will be provided from a local insurance company. The company undertakes to indemnify the institution (NMIMR) against any claims for negligent and non-negligent harm to any volunteer as a result of his/her participation in the study. Coverage will continue for 3 years from the time of enrolment.

14.6 Subject Confidentiality

Subject confidentiality is strictly held in trust by the investigators, the staff, and the sponsor and their agents. This confidentiality is extended to cover testing of biological samples in addition to the clinical information relating to participating subjects.

The study protocol, documentation, data and all other information generated will be held in strict confidence and will be kept in a locked cabinet. Only study personnel will have access to these

records. No information concerning the study or the data will be released to any unauthorized third party, without prior written approval of the sponsor.

The study monitor or other authorized representatives of the sponsor may inspect all documents and records required to be maintained by the investigator, including but not limited to, medical records and pharmacy records for the subjects in this study. The clinical study site will permit access to such records.

14.7 Study Discontinuation

Upon study discontinuation, no crossover studies are planned at this point. Only SAEs and pregnancies will be followed to resolution.

15 DATA HANDLING AND RECORD KEEPING

The investigator is responsible to ensure the accuracy, completeness, legibility, and timeliness of the data reported. All source documents should be completed in a neat, legible manner to ensure accurate interpretation of data. Black or blue ink is required to ensure clarity of reproduced copies. When making changes or corrections, cross out the original entry with a single line, and initial and date the change. DO NOT ERASE, OVERWRITE, OR USE CORRECTION FLUID OR TAPE ON THE ORIGINAL.

Data collection forms derived from the eCRF will be provided for use as source documents and maintained for recording data for each subject enrolled in the study. Data reported in the eCRF derived from source documents should be consistent with the source documents or the discrepancies should be explained

DMID and/or it designee will provide guidance to investigators on making corrections to the source documents and eCRF.

15.1 Data Management Responsibilities

All source documents and laboratory reports must be reviewed by the clinical team and data entry staff, who will ensure that they are accurate and complete. Adverse events must be graded, assessed for severity and causality, and reviewed by the site PI or designee.

Data collection is the responsibility of the clinical trial staff at the site under the supervision of the site PI. During the study, the investigator must maintain complete and accurate documentation for the study.

The EMMES Corporation will serve as the SDCC for this study and will be responsible for data management, quality review, analysis, and reporting of the study data.

15.2 Data Capture Methods

Clinical data (including AEs, concomitant medications, and reactogenicity data) and clinical laboratory data will be entered into a 21 CFR Part 11-compliant data entry system provided by the EMMES Corporation. The data system includes password protection and internal quality

checks, such as automatic range checks, to identify data that appear inconsistent, incomplete, or inaccurate. Clinical data will be entered directly from the source documents.

15.3 Types of Data

Safety assessments will be based on solicited reactogenicity reports collected from review of memory aids, AE and SAE reports. Reactogenicity will be summarized by severity, across the entire observation period and into local and systemic symptoms. All AEs will be MedDRA® coded for preferred term and system organ class. The rate of AEs in aggregate, and by MedDRA codes, will be computed for each vaccine group.

The number of SAEs is likely to be small in this study and will be reported by a detailed listing showing the type, MedDRA coding, relevant dates (vaccination and AE), severity and outcome for each event. The list will be by vaccine group. In the event that the number of all AEs is small, they will also be listed with the additional attribution of seriousness.

15.4 Data Requirements

An individual forms grid, which indicates the current status of forms submission for each subject, is provided as part of the EMMES Corporation AdvantageEDC℠ internet data entry system.

15.5 Study Records Retention

Study documents should be retained for a minimum of 2 years after the last approval of a marketing application in an ICH region and until there are no pending or contemplated marketing applications in an ICH region or at least 2 years have elapsed since the formal discontinuation of clinical development of the investigational product. These documents should be retained for a longer period, however, if required by local regulations. No records will be destroyed without the written consent of the sponsor, if applicable. It is the responsibility of the sponsor to inform the investigator when these documents no longer need to be retained.

15.6 Protocol Deviations

Protocol deviations will be documented. Protocol deviation data will be entered at NMIMR into The EMMES Corporation's AdvantageEDC℠.

A protocol deviation is any noncompliance with the clinical trial protocol, GCP, or manual of procedures requirements. The noncompliance may be either on the part of the subject, the investigator, or the study site staff. As a result of deviations, corrective actions are to be developed by the site and implemented promptly.

These practices are consistent with ICH E6:

4.5 Compliance with Protocol, Sections 4.5.1, 4.5.2, and 4.5.3

5.1 Quality Assurance and Quality Control, Section 5.1.1

5.20 Noncompliance, Sections 5.20.1, and 5.20.2.

It is the responsibility of the site to use continuous vigilance to identify and report deviations within 5 working days after identification of the protocol deviation, or within 5 working days after the scheduled protocol-required activity. All deviations must be promptly reported to the DMID, via the EMMES Corporation

All deviations from the protocol must be addressed in study subject source documents. A completed copy of the DMID protocol deviation form (The EMMES Corporation form) must be maintained in the regulatory file, as well as in the subject's source document workbook. Protocol deviations must be sent to the local IRB per their guidelines. The site PI/study staff is responsible for knowing and adhering to their IRB requirements.

16 PUBLICATION POLICY

All investigators funded by the NIH must submit or have submitted for them to the National Library of Medicine's PubMed Central an electronic version of their final, peer-reviewed manuscripts upon acceptance for publication, to be made publicly available no later than 12 months after the official date of publication. The NIH Public Access Policy ensures the public has access to the published results of NIH funded research. It requires investigators to submit final peer-reviewed journal manuscripts that arise from NIH funds to the digital archive PubMed Central upon acceptance for publication. Further, the policy stipulates that these papers must be accessible to the public on PubMed Central no later than 12 months after publication.

Publication and authorship policies will be clearly outlined in this section, and should be consistent with the specific contract, grant and/or Clinical Trials Agreements. Policies regarding sub-studies should be outlined in this section.

Refer to:

http://publicaccess.nih.gov/
http://grants.nih.gov/grants/guide/notice-files/NOT-OD-08-033.html

17 LITERATURE REFERENCES

1. Sim BKL, Chitnis CE, Wasniowska K, et al. Receptor and ligand domains for invasion of erythrocytes by Plasmodium falciparum. Science 1994;264:1941-4.
2. Liang H and Sim BKL. Conservation of structure and function of the erythrocyte-binding domain of Plasmodium falciparum EBA-175. Mol Biochem Parasitol 1997;84:241-5.
3. Jones TR, Narum DL, Gozalo AS, et al. Protection of Aotus monkeys by Plasmodium falciparum EBA-175 region II DNA prime-protein boost immunization regimen. J Infect Dis 2001;183:303-12.

Appendix A: Schedule of Procedures

**Study Visit**	**1**	**2**	**3**	**4**	**5**	**6**	**7**	**8**	**9**	**10**	**11**	**12**	**13**	**14**	**15**	**16**
**Study Day**<br>Window	**Screen**[1]	**0**	**2**<br>**±1**	**7**<br>**±2**	**14**<br>**±3**	**28**<br>**±3**	**30**<br>**±1**	**35**<br>**±1**	**42**<br>**±3**	**180**<br>**±14**	**182**<br>**±1**	**187**<br>**±2**	**194**<br>**±3**	**208**<br>**±7**	**258**<br>**±21**	**348**<br>**±21**
Review Inclusion/Exclusion Criteria	X	X				X				X						
Obtain Informed Consent	X															
Medical History	X	X	X	X	X	X	X	X	X	X	X	X	X	X		
Targeted Physical Examination[2]	X	X	X	X	X	X	X	X	X	X	X	X	X	X		
Vital Signs[3]	X	X	X	X	X	X	X	X	X	X	X	X	X	X	X	X
Clinical Safety Laboratory Testing[4]	X	X			X	X			X	X			X	X		
Urine Pregnancy Test[5]		X				X				X				X		
HIV, HCV, HBsAg, (0.5mL)	X															
Blood for Immunogenicity[7]		X			X	X			X	X			X			
Concomitant Medications	X	X	X	X	X	X	X	X	X	X	X	X	X	X		
Vaccination		X				X				X						
Assessment of Reactogenicity[8]		X	X	X	X	X	X	X	X	X	X	X	X	X		
Adverse Event Assessment		X	X	X	X	X	X	X	X	X	X	X	X	X		
Distribute Memory Aid[9]		X				X				X						
Review/Collect Memory Aid			X	X	X		X	X	X		X	X	X			
Blood Sample Volume (230 mL)	10	35	0	0	35	35	0	0	35	35	0	0	35	10		
SAE assessment		X	X	X	X	X	X	X	X	X	X	X	X	X	X	X

[1] Screening activities must be completed within 28 days of Day 0.

[2] A physical examination that assesses lymph nodes, lungs, abdomen and heart will be performed on the screening visit, and thereafter if indicated by the subject's interim history. Height and weight will be measured on the screening visit.

[3] Temperature will be taken on visits 1 through 16. Blood pressure and pulse will be taken on visits 1, 2, 6 and 10.

[4] Clinical safety laboratory profile includes: Hematology: (4.5 mL EDTA): CBC with platelet count (total WBC count and differential, Hgb, hematocrit) ; chemistry (5.0 mL SST): serum creatinine, electrolytes, ALT, AST, plasma glucose; 0.5ml for serology test kits and urinalysis (10mL random void). Abnormal values will be followed until resolution and supplemental laboratory testing will be performed, as appropriate, to ascertain the diagnosis. A thick blood smear will be done and stored using blood drawn for safety labs and immunology.

[5] Urine pregnancy tests will be required for female subject of childbearing potential; must be less than 48 hours prior to vaccination.

[7] Immunogenicity-sera will be collected for humoral immune responses (25-mL SST),

[8] Reactogenicity events will be collected after vaccination through Day 14 and followed until resolution. An examination of the vaccination site will be assessed through Day 208. Subjects will be reminded to notify the study center if they develop an oral temperature of 37.8 degrees or higher during the week after vaccination, or if they develop any severe reactions during the study.

[9] Subjects will be provided with a memory aid, clear plastic ruler, and a digital thermometer, and will be instructed in their use prior to discharge from the clinic.

Appendix B: Laboratory Adverse Event Grading Scale

Parameter	Grade 0 "Normal" Screening Values	Grade 1 Mild	Grade 2 Moderate	Grade 3 Severe
Hemoglobin (g/dL)	>10.5 (F)<br>>12.5 (M)	9.5 - 10.5 (F)<br>11 - 12.5 (M)	8.5 - 9.4 (F)<br>10 - 10.9 (M)	≤8.4 (F)<br>≤9.9(M)
WBC (cells/µL)<br>Decreased<br>Increased	3401<br>8999	2500 - 3400<br>9000 - 14000	2000 - 2499<br>14001 - 19000	<2000<br>≥19001
Platelet count Decreased (per µL)	≥125K	100K - 124K	50K - 99K	<50K
AST (IU/L)	≤ 65 (M)<br>≤ 47 (F)	65 < AST ≤ 163 (M)<br>47< AST ≤ 118 (F)	163 < AST ≤ 260 (M)<br>118 < AST ≤ 188 (F)	> 260 (M)<br>> 188 (F)
ALT (IU/L)	≤ 54 (M)<br>≤ 40 (F)	54 < ALT ≤ 135 (M)<br>40 < ALT ≤ 100 (F)	135 < ALT ≤ 216 (M)<br>100 < ALT ≤ 160 (F)	> 216 (M)<br>> 160 (F)
Creatinine (**umol/L**	≤ 141 (M)<br>≤ 121 (F)	141 < Cr ≤ 211(M)<br>121 < Cr ≤ 182 (F)	211< Cr ≤282 (M)<br>182 < Cr ≤ 242(F)	> 283 (M)<br>> 242(F)
Urinalysis*<br>Protein<br>Blood<br>WBC	0-trace<br>0-trace<br>0-5	1+<br>1+<br>6-10	2+<br>2+<br>11-50	3+<br>3+<br>>50
Non-Fasting Blood Glucose** mg/dl<br>Increased<br>Decreased	69-110	110-125<br>65-69	126-200<br>55-64	>200<br><45
Fasting Blood Sugar mg/dL**	69-100	100-110	111-125	>125
Sodium mEq/L<br>Increased<br>Decreased	135-148	149-153<br>131-134	154-157<br>127-130	>157<br><127
Potassium mEq/L<br>Increased<br>Decreased	3.5-5.5	5.6-5.8<br>3.1-3.4	5.9-6.2<br>2.7-3.0	>6.2<br><2.7

Koram KA, Addae MM**, Ocran JC, Adu-Amankwa S, Rogers WO, Nkrumah FK** Population based reference intervals for common blood haematological and biochemical paramemeters in the Akuapem North district. *Ghana Med J* 2007 Dec; 41(4):160-166 (All parameters are taken from this publication except for Non-Fasting and Fasting Blood Sugars which are from the FDA's "**Guidance for Industry; Toxicity Grading Scale for Healthy Adult and Adolescent Volunteers Enrolled in Preventive Vaccine Clinical Trials)**

*For women, results apply only if not menstruating.

** Random plasma glucose will be drawn on volunteers. Elevated glucose will be confirmed by repeat fasting plasma glucose . Elevated Random plasma glucose levels on Study Day s 0, 28 and 180 will be evaluated with a FBS on Study Day 14, 42 and 194 respectively.

F = Female, M = Male

ALT = alanine aminotransferase, AST = aspartate aminotransferase

WBC = white blood cell

Appendix C: Reactogenicity Grading Scale

Local Reaction	Mild (Grade 1)	Moderate (Grade 2)	Severe (Grade 3)
Pain	Does not interfere with activity	Interferes with activity	Prevents daily activity
Tenderness	Mild pain to touch	Pain with movement	Significant pain at rest
Erythema*	> 0 - <30 mm	≥ 30 - <120 mm	≥120 mm
Edema*	> 0 - <30 mm	≥ 30 - < 120 mm	≥120 mm
Induration*	>0 - <15 mm	15 - 30 mm	>30 mm

* Measure at greatest single diameter.
