# Supplementary material for: Safety and Immunogenicity of EBA-175 RII-NG Malaria Vaccine Administered Intramuscularly in Semi-Immune Adults: A Phase 1, Double-Blinded Placebo Controlled Dosage Escalation Study
Source: PLoS One. 2016 Sep 19;11(9):e0163066. doi: 10.1371/journal.pone.0163066 (PMC5028127; doi:10.1371/journal.pone.0163066)
Supplement: S1 Table — Population based reference intervals for common blood haematological and biochemical parameters; Koram et al., 2007 [39]. For women, results apply only if not menstruating. ** Random plasma glucose was drawn on volunteers. Elevated glucose was confirmed by repeat fasting plasma glucose. F: Female; M: Male; ALT: alanine aminotransferase; AST: aspartate aminotransferase; WBC: white blood cell; ULN: upper limit of normal (DOC) [file pone.0163066.s003.doc]

**S1 Table**. Laboratory adverse event grading scale

| **Parameter** | **Grade 0**  **“Normal” Screening Values** | **Grade 1**  **Mild** | **Grade 2**  **Moderate** | **Grade 3**  **Severe** |
| --- | --- | --- | --- | --- |
| **Hemoglobin (g/dL)** | >10.5 (F) | 9.5 - 10.5 (F) | 8.5 - 9.4 (F) | ≤8.4 (F) |
|  | >12.5 (M) | 11 - 12.5 (M) | 10 - 10.9 (M) | ≤9.9(M) |
| **WBC (cells/µL)** | | | | |
| Decreased | < 3401 | 2500 - 3400 | 2000 - 2499 | <2000 |
| Increased | >8999 | 9000 - 14000 | 14001 - 19000 | >19001 |
| **Platelet count (per µL)** | >125K | 100K - 124K | 50K - 99K | <50K |
| **AST (IU/L)** | <1 x ULN | >1 - 2.5 x ULN | >2.5 - 4 x ULN | >4 x ULN |
| **ALT (IU/L)** | <1 x ULN | >1 - 2.5 x ULN | >2.5 - 4 x ULN | >4 x ULN |
| **Creatinine (mg/dL)** | <1 x ULN | >1 -1.5 x ULN | >1.5 - 2 x ULN | >2 x ULN |
| **Urinalysis*** | | | | |
| Protein | 0-trace | 1+ | 2+ | 3+ |
| Blood | 0-trace | 1+ | 2+ | 3+ |
| WBC (per hpf) | 0-5 | 6-10 | 11-50 | >50 |
| ****Glucose mg/dL** | | | | |
| Increased | 65-109 | 110-125 | 126-140 | >140 |
| Decreased |  | 50-64 | 40-49 | <40 |
| **Sodium mEq/L** | | | | |
| Increased | 135-148 | 149-153 | 154-157 | >157 |
| Decreased |  | 131-134 | 127-130 | <127 |
| **Potassium mEq/L** | | | | |
| Increased | 3.5-5.5 | 5.6-5.8 | 5.9-6.2 | >6.2 |
| Decreased |  | 3.1-3.4 | 2.7-3.0 | <2.7 |

Population based reference intervals for common blood haematological and biochemical parameters; Koram *et al.,* 2007 [39].

For women, results apply only if not menstruating.

** Random plasma glucose was drawn on volunteers. Elevated glucose was confirmed by repeat fasting plasma glucose. F: Female; M: Male; ALT: alanine aminotransferase; AST: aspartate aminotransferase; WBC: white blood cell; ULN: upper limit of normal
